# Microglial Interactions with Synapses Are Modulated by Visual Experience

**DOI:** 10.1371/journal.pbio.1000527

**Published:** 2010-11-02

**Authors:** Marie-Ève Tremblay, Rebecca L. Lowery, Ania K. Majewska

**Affiliations:** Department of Neurobiology and Anatomy and Center for Visual Science, University of Rochester, Rochester, New York, United States of America; University of Pennsylvania, United States of America

## Abstract

Microglia are the immune cells of the brain. In the absence of pathological insult, their highly motile processes continually survey the brain parenchyma and transiently contact synaptic elements. Aside from monitoring, their physiological roles at synapses are not known. To gain insight into possible roles of microglia in the modification of synaptic structures, we used immunocytochemical electron microscopy, serial section electron microscopy with three-dimensional reconstructions, and two-photon in vivo imaging to characterize microglial interactions with synapses during normal and altered sensory experience, in the visual cortex of juvenile mice. During normal visual experience, most microglial processes displayed direct apposition with multiple synapse-associated elements, including synaptic clefts. Microglial processes were also distinctively surrounded by pockets of extracellular space. In terms of dynamics, microglial processes localized to the vicinity of small and transiently growing dendritic spines, which were typically lost over 2 d. When experience was manipulated through light deprivation and reexposure, microglial processes changed their morphology, showed altered distributions of extracellular space, displayed phagocytic structures, apposed synaptic clefts more frequently, and enveloped synapse-associated elements more extensively. While light deprivation induced microglia to become less motile and changed their preference of localization to the vicinity of a subset of larger dendritic spines that persistently shrank, light reexposure reversed these behaviors. Taken together, these findings reveal different modalities of microglial interactions with synapses that are subtly altered by sensory experience. These findings suggest that microglia may actively contribute to the experience-dependent modification or elimination of a specific subset of synapses in the healthy brain.

## Abstract 2

Microglia, the brain's immune cells, show unique interactions with nearby synaptic elements under non-pathological conditions that are sensitive to changes in sensory experience.

## Author Summary

Microglia are important players in immune responses to brain injury. In the event of pathological insults, microglia rapidly become activated and acquire the ability to release various inflammatory molecules that influence neuronal survival as well as synaptic function and plasticity. Similarly to macrophages in other areas of the body, activated microglia can engulf, or phagocytose, cellular debris and are believed to eliminate synapses. In the absence of pathological insult, microglia are more quiescent, but still, these immune surveillants continually sample their surrounding environment and contact neighboring cells and synapses. To further explore the roles of microglia at synapses under non-pathological conditions, we used quantitative electron microscopy and two-photon in vivo imaging to characterize the interactions between quiescent microglia and synaptic elements in the visual cortex of juvenile mice. We also examined the “activity-dependent” processes involved by preventing light exposure in a group of mice. We show surprising changes in microglial behavior during alterations in visual experience, such as increased phagocytosis of synaptic elements and interaction with subsets of structurally dynamic and transient synapses. These observations suggest that microglia may participate in the modification or elimination of synaptic structures, and therefore actively contribute to learning and memory in the healthy brain.

## Introduction

Upon invasion of the central nervous system during embryonic and early postnatal development, bone-marrow-derived microglia become involved in apoptosis and phagocytic elimination of supernumerary neurons [1]–[3]. As they complete their differentiation, microglia change their morphology from amoeboid to ramified and are thought to become quiescent [4]. In the event of pathological insult, microglia rapidly become activated, thicken and retract their processes, migrate to the site of injury, proliferate, and participate in the presentation of antigens, phagocytosis of cellular debris, and secretion of proteases that promote microglial motility, as well as myelin and extracellular matrix degradation [5]–[7]. Additionally, activated microglia can separate presynaptic axon terminals from postsynaptic neuronal perikarya or dendrites in a process known as synaptic stripping [8].

Even though microglia are quiescent under non-pathological conditions, their highly motile processes continually survey the local environment and make transient contacts with astrocytes, neuronal perikarya, axon terminals, and dendritic spines in vivo [9]–[11]. Microglial apposition with astrocytic and neuronal elements has also been observed with electron microscopy (EM) in situ [9], but a detailed analysis of microglial ultrastructural relationships is still lacking. Reports of spontaneous engulfment of cellular debris [10] suggest that resting microglia may exert phagocytic roles in the healthy brain. Because changes in the level of neuronal activity can also modify the volume of neuropil that microglia sample [10], as well as their frequency of contact with axon terminals [9], Wake et al. [9] proposed that resting microglia could monitor the functional state of synapses. However, aside from immune surveillance, the fate of synaptic architecture under the care of microglia remains poorly understood. The dynamic nature of microglial processes and their interaction with synapses suggest that microglia could effect structural changes at synapses, which are crucial to circuit remodeling and brain plasticity.

To begin to investigate this possible task of quiescent microglia at synapses, we verified whether microglial interactions with synapses occur at random or coincide with structural synaptic changes and alterations in sensory experience. Specifically, we characterized the ultrastructural and structural/dynamic interactions between microglia and synapse-associated elements during normal sensory experience, sensory deprivation, and subsequent light exposure in the primary visual cortex (V1) of juvenile mice. In addition to revealing the three-dimensional (3-D) geometry of cell–cell contacts between microglia and all synapse-associated elements (dendritic spines, axon terminals, perisynaptic astrocytic processes, and synaptic clefts), our observations uncovered new modes of microglia–synapse interaction under non-pathological conditions, particularly the regulation of the perisynaptic extracellular space and the phagocytosis of synaptic elements. Moreover, we found that microglia specifically localize to the vicinity of a subset of synaptic elements in vivo, in particular the structurally dynamic and transient dendritic spines. Lastly, we demonstrate that several modalities of microglia–synapse interactions are regulated by sensory experience. Thus, our findings indicate that microglia are not activated only during early brain development or pathological conditions; rather, they also subtly change their behavior toward synapses in correspondence with sensory experience. This raises the intriguing possibility that microglia may contribute to fine-tuning the plastic capacities of individual synapses in the healthy brain.

## Results

### Ultrastructural Relationships between Microglia and Synapses

**Figure 1:**
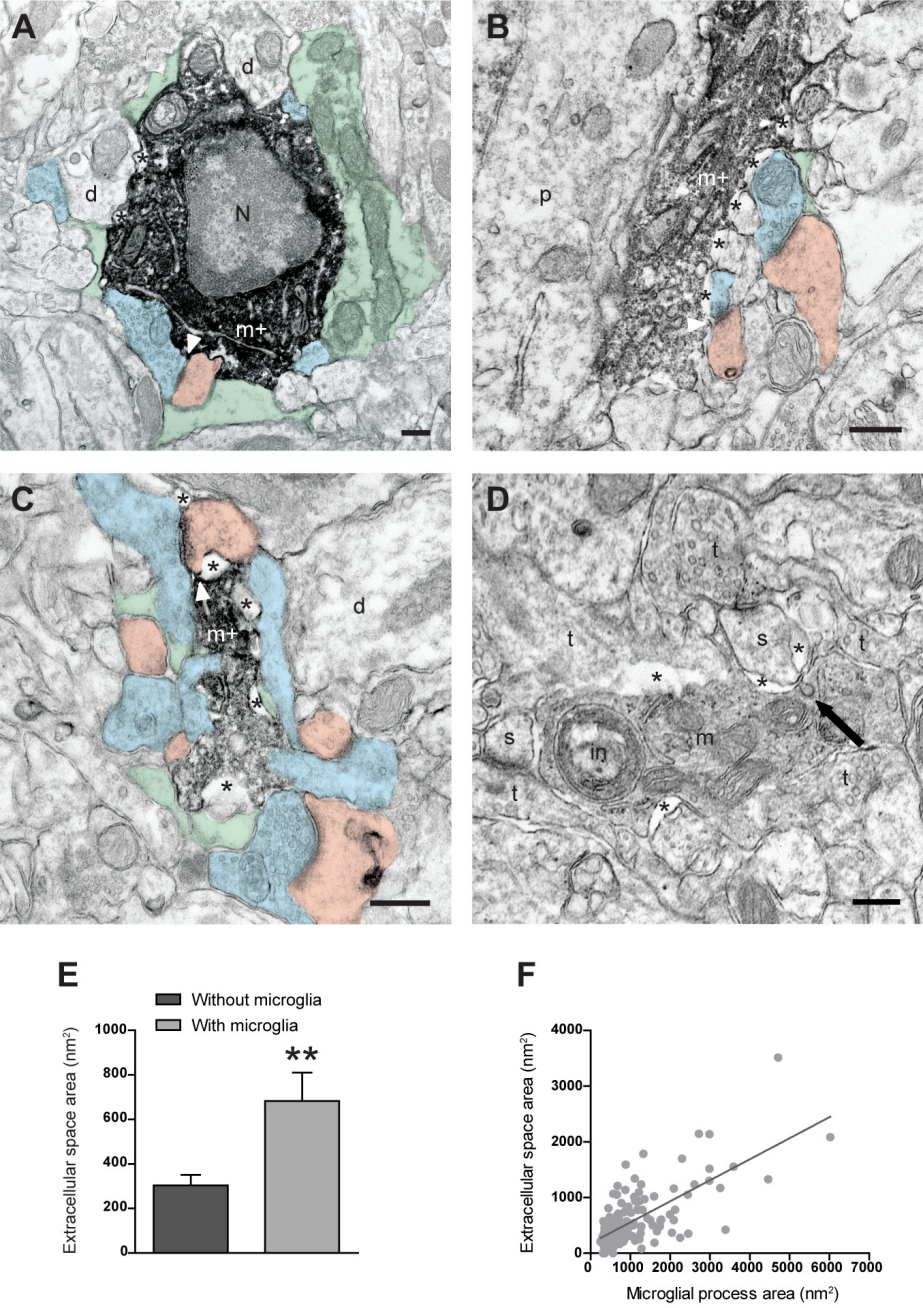
Ultrastructural interactions between microglia and synapses during normal sensory experience. (A–C) EM images showing IBA1-immunostained microglial (m+) cell bodies (A), as well as large (B) and small (C) processes, surrounded by extended extracellular space (asterisks) and contacting axon terminals (blue), dendritic spines (pink), perisynaptic astrocytes (green), and synaptic clefts (arrowheads). d, dentrite; N, nucleus; p, perikaryon. Scale bars = 250 nm. (D) EM image showing extended microglia-associated extracellular spaces (asterisks) after glutaraldehyde instead of acrolein fixation. The unlabeled microglial process (m) makes direct contacts with dendritic spines (s) and axon terminals (t), and displays an inclusion (in), as well as a clathrin-coated pit (black arrow) at the site of contact with a spine. Scale bar = 250 nm. (E) Extracellular space areas with or without contact with IBA1-positive microglial process (mean ± SEM). **, *p*<0.01. (F) Correlation between the areas of microglial processes and associated extracellular space (*r* = 0.48; *p*<0.0001). See also Figure S1 and Tables S1 and S2.

To provide a detailed view of the modes of interaction between microglia and excitatory synapses, we analyzed their ultrastructural relationships in layer II of mouse V1 on postnatal day (P) 28, around the peak of the critical period for experience-dependent plasticity [12]. Using immunocytochemical EM with an antibody against the microglia-specific marker IBA1 [13] (see Figure S1 for IBA1 immunostaining at the light microscopic level), we found that microglial cell bodies, as well as proximal and distal processes, juxtaposed synapse-associated elements including synaptic clefts (Figures 1A–1C), an area generally thought to be exclusively reserved for astrocytic processes. Quantitative analysis revealed that the vast majority of microglial process profiles directly contacted at least one of the synapse-associated elements (synaptic index: 94%±0.6%; ∼1,000 µm^2^ of neuropil in each of three animals). Axon terminals, dendritic spines, perisynaptic astrocytic processes, and synaptic clefts were contacted by microglial processes, in decreasing order of frequency (*n* = 150 IBA1-positive microglial processes; three animals; see Table S1 for detailed analysis), and more than one synapse-associated element was generally contacted by each process (68%±4%; see Table S2).

**Figure 2:**
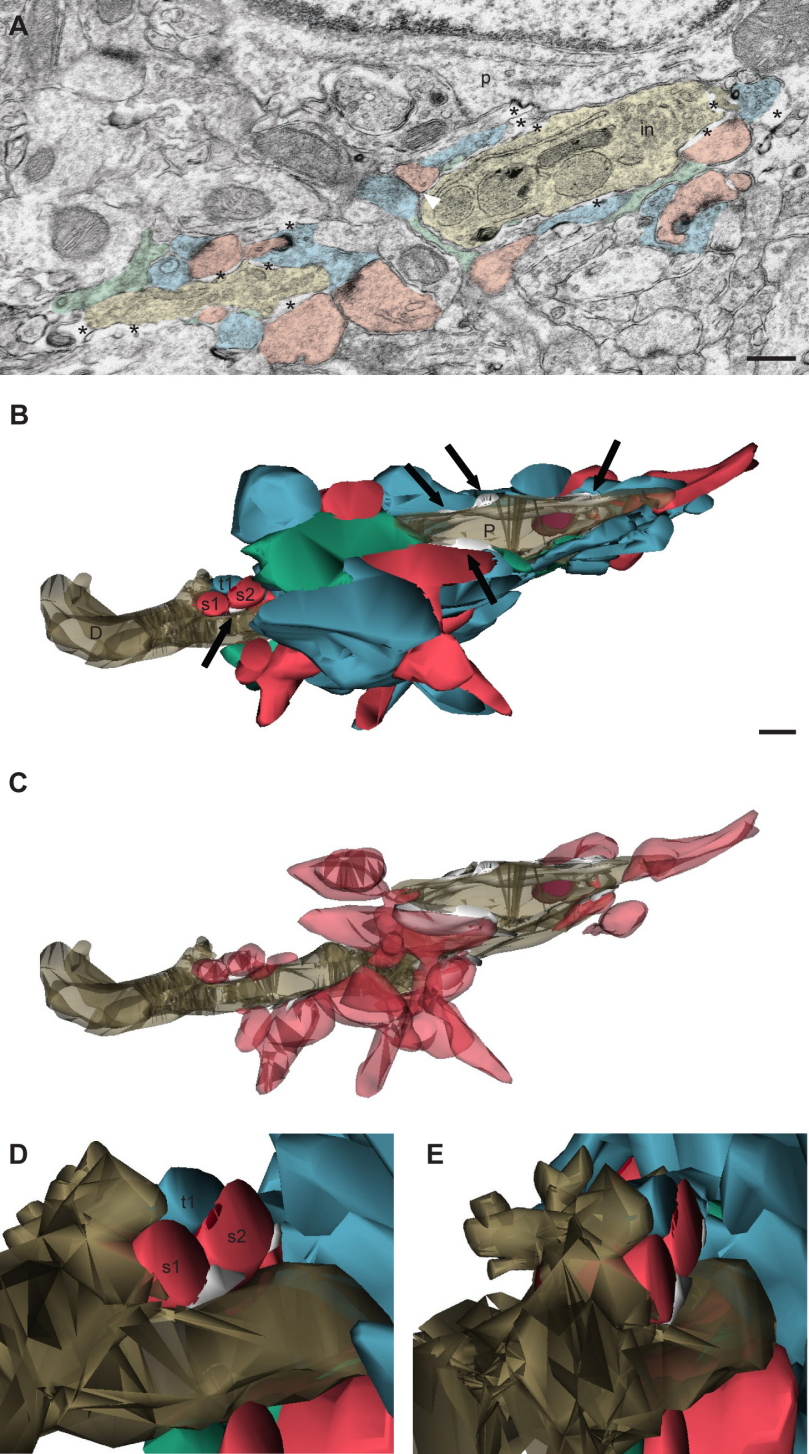
3-D reconstructions of the ultrastructural interactions between microglia and synapses during normal sensory experience. (A) SSEM image showing a microglia (beige) contiguous to a neuronal perikaryon (p), with its associated extracellular space (asterisks) and contacted synapse-associated elements. Color scheme as in Figure 1. in, inclusion. (B) Partial 3-D reconstruction of the microglial proximal process (P; taupe) cut in transverse. Purple indicates the inclusion. Both processes simultaneously contact multiple axon terminals (blue), dendritic spines (red), and perisynaptic astrocytic processes (green), and are distinctively surrounded by extracellular space pockets of various size and shape (white; black arrows). (C) Additional view showing only microglia, dendritic spines, and extracellular space. (D and E) Insets illuminating the 3-D geometry of the distal protrusion and its structural relationships with one axon terminal (t1), two dendritic spines (s1 and s2; postsynaptic density in dark red), and a pocket of extracellular space (white), which are partially reconstructed. For clarity, an astrocytic process was removed from the scene. Scale bars = 250 nm. See also Figures S2, S3, S4 and Table S3.

To uncover the 3-D relationships between microglia and synapse-associated elements, we used serial section EM (SSEM) with 3-D reconstructions (Figures 2 and S2) in layer II of mouse V1 at P28. The 3-D reconstructions showed that proximal and distal microglial processes simultaneously contacted synapses of different shape and size, with typically more than one subcellular element contacted at individual synapses (Figures 2B, 2D, 2E, S2A, and S2B). Whereas most contacts between microglia and synapses occurred en passant along microglial processes, without morphologic evidence of specialization, strikingly, the reconstructed distal microglial process displayed finger-like protrusions that wrapped around a dendritic spine making a synapse with an axon terminal (Figure 2B, 2D, and 2E). Interestingly, the proximal microglial process was found to engulf cellular components (see SSEM images in Figure S4, but also the reconstructed cellular inclusion in Figures 2B, 2C, and S2A, S2B, S2C), suggesting microglial involvement in phagocytosis during normal sensory experience, along with our immunocytochemical EM observations that microglial perikarya and large processes sometimes contained cellular inclusions. Lastly, analysis of the series also showed occasional coated pits at the sites of cell–cell contact between microglia and dendritic spines, axon terminals, or astrocytic processes, either inside microglia or inside synapse-associated elements (see Figures 1D and S3 for examples).

### Distinctive Properties of the Microglia-Associated Extracellular Space

Immunocytochemical EM and SSEM also revealed the appearance of large electron-lucent extracellular spaces nearby microglia, with both acrolein (Figures 1A–1C and 2A) and glutaraldehyde (Figure 1D) fixatives. Pockets of extracellular space, which consists of interstitial fluid supplemented with various extracellular matrix proteins, were otherwise rarely observed around any other structural elements in juvenile mice, in clear contrast with early postnatal stages of development [14]–[16]. Areas of extracellular space apposing IBA1-positive microglia were found to be significantly larger (985±41 nm^2^; *n* = 3 animals; ∼500 µm^2^ of neuropil in each) than areas not associated with IBA1-positive processes (382±18 nm^2^; *p*<0.0002; Figure 1E). This analysis likely underestimated the association of extracellular space with microglial processes because of the partial penetration of antibodies under these stringent immunocytochemical conditions. In fact, most extracellular spaces were associated with unlabeled structural elements that resembled microglia morphologically (see Materials and Methods for identification criteria). In addition, areas of a microglial process and associated extracellular space were tightly correlated (*r* = 0.48; *p*<0.0001; *n* = 150 processes; three animals; Figure 1F), suggesting that microglia may exert a large influence in the creation of such space. SSEM with 3-D reconstructions also showed that microglia-associated spaces exhibited highly complex and varied morphologies (Figures 2B, 2C, and S2C). Quantitative analysis of the series revealed that the 3-D pockets of extracellular space varied in volume by two orders of magnitude: from 20,223 to 7,048,921 nm^3^ (mean: 905,834 nm^3^; median: 150,225 nm^3^; *n* = 15 extracellular spaces; Table S3). In light of these results, when considering the interactions of synapses and microglia, it is important to take into account the complex organization of extensive contacts between a single microglia and every synapse-associated element at multiple synapses, interrupted by many pockets of geometrically complex microglia-specific extracellular spaces, simultaneously throughout the neuropil.

Taken together, our findings from immunocytochemical EM and SSEM with 3-D reconstructions indicate that microglia are uniquely positioned to play several physiological roles at synapses: through cell–cell communication with perisynaptic astrocytic processes, dendritic spines, and axon terminals simultaneously, as well as through the regulation of the extracellular environment intervening between them.

### Dynamics of the Structural Relationships between Microglia and Synapses

To characterize the structural dynamics of microglia–synapse interactions, we used two-photon in vivo imaging of layers I/II of V1 in juvenile CX_3_CR1-GFP/Thy1-YFP mice [17],[18], in which both microglia and layer V neurons are fluorescently labeled. We utilized a thinned-skull preparation, which minimizes brain injury and allows long-term tracking of microglial dynamics without causing microglial activation [19] (Figure S5). Even though the resolution of two-photon microscopy (see Materials and Methods for details on measurement of the experimental point spread function) prevents visualization of direct contacts between fluorescently labeled elements, it enables the study of their structural dynamics and determination of close proximity (the apparent colocalization of fluorescence for microglial and neuronal elements was considered putative contact). A recent study demonstrated a relatively constant duration (4.60±0.08 min) of putative contacts between microglia and synaptic elements in layer II/III of juvenile mouse V1 [9]. In contrast, our time-lapse imaging (every 5 min for 30–120 min) revealed similar contacts between microglial processes and a subset of the YFP-labeled dendritic spines and axon terminals (*n* = 37 putative contacts with spines and 29 putative contacts with terminals in eight animals; Figures S7A and 3A; see Videos S1 and S4) that varied between 5 and 50 min. The cortical layers examined or the identity of synapses imaged (GFP-M and YFP-H mice label different subsets of pyramidal cells) [20]–[23] might explain this discrepancy. Nevertheless, these differences in contact duration reveal a new dimension in microglial interactions with synapses in the healthy brain.

**Figure 3:**
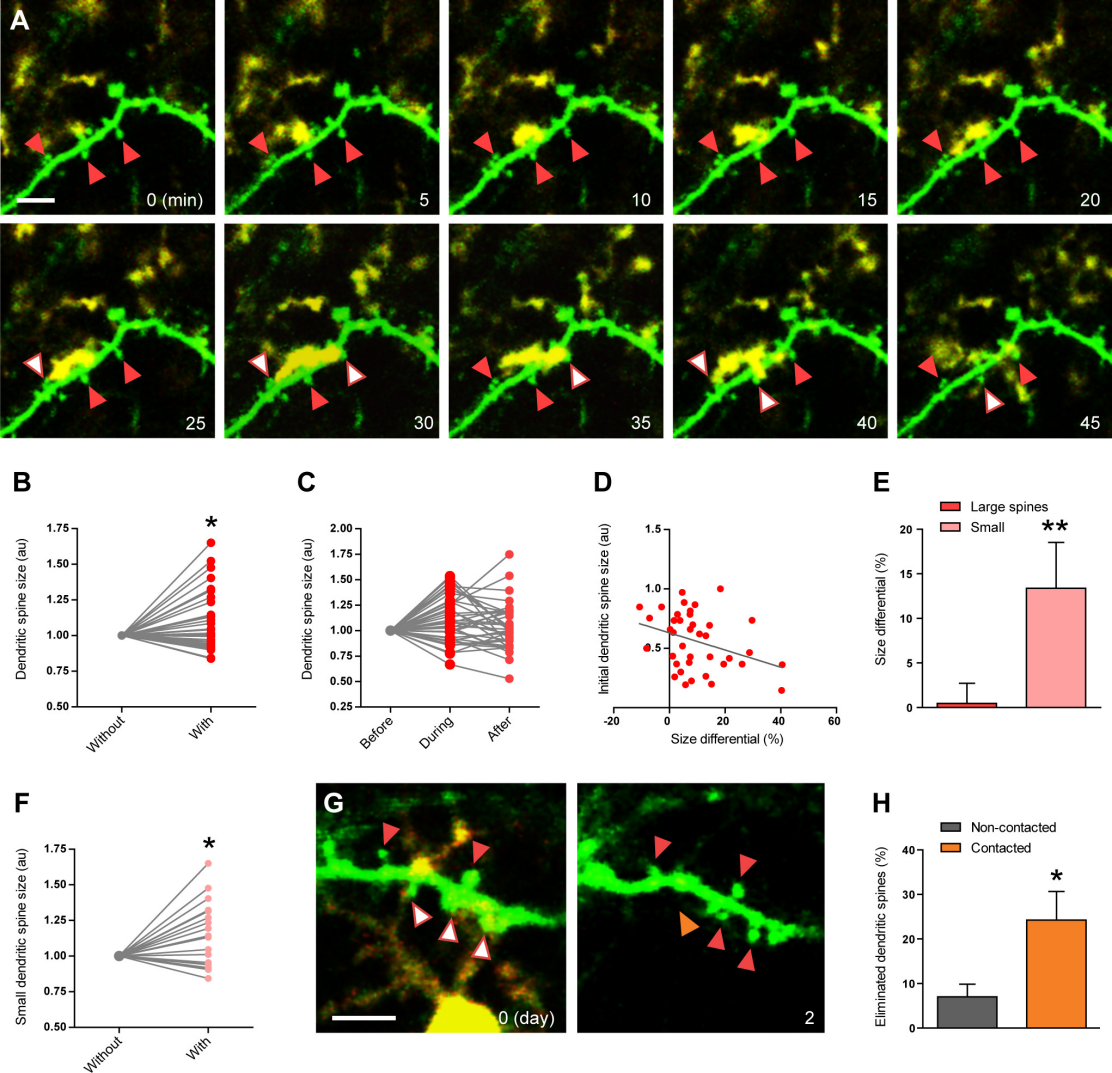
Structural/dynamic interactions between microglia and dendritic spines during normal sensory experience in vivo. (A) Time-lapse images showing three dendritic spines (green; red arrowheads) contacted by microglia (yellow; white arrowheads) over 45 min. Scale bar = 5 µm. (B) Dendritic spine size changes during microglial contact. (C) Dendritic spine size before, during, and after contact. (D) Correlation between the initial dendritic spine size and the change in spine size during contact. (E) Average size changes in the presence versus in the absence of microglial contact for large and small dendritic spines (mean ± SEM). (F) Change in small dendritic spine size during microglial contact. (G) Images from chronic experiments showing the elimination of a dendritic spine that had been contacted by a microglial process over 2 d (white arrowhead during the contact; orange arrowhead after the contact), while the other contacted (white arrowheads during the contact; red arrowheads after the contact) and non-contacted (red arrowheads) spines remain stable. Scale bar = 5 µm. (H) Proportion of dendritic spines eliminated over 2 d in spine populations contacted and not contacted by microglia during the first imaging session (mean ± SEM). Values in (B), (C), and (F) were normalized to the first condition, and values in (D) were normalized to the size of the largest spine. au, arbitrary units. *, *p*<0.05; **, *p*<0.01. See also Figures S5, S6, S7, S8 and Videos S1 and S4.

To verify whether microglial processes target specific subsets of synapses, we measured the size of dendritic spines and axon terminals in the presence and absence of a putative microglial contact. In this analysis, we noticed that dendritic spines that were in close proximity to microglial processes at any point during the imaging session were generally smaller than the rest of the spine population. We quantitatively recorded spine size at the beginning of imaging and found that dendritic spines receiving putative contact during imaging were significantly smaller than spines remaining non-contacted (*p*<0.001; *n* = 31 contacted spines in five animals and 45 non-contacted spines in three animals, with infrequent stubby spines excluded; see Materials and Methods). When microglia came into close proximity with these synaptic structures, individual pre- and postsynaptic elements both expanded and shrank: 38% of axon terminals grew, 55% shrank, and 7% remained stable (Figure S7B and S7C). Average axon terminal sizes were not significantly different with and without putative microglial contact (size differential: −1%±3%; *p*>0.9; *n* = 24 terminals and 29 putative contacts in three animals; Figure S7B and S7C), nor correlated to initial terminal size or putative microglial contact duration (*p*>0.5 and *p*>0.2, respectively; Figure S7D and S7E). In contrast, 62% of dendritic spines grew, 32% shrank, and 6% remained stable during putative contact; additionally, we observed a significant increase in average spine sizes in the presence versus in the absence of putative microglial contact (size differential: 9%±3%; *p*<0.03; *n* = 31 spines and 37 contacts in five animals; Figure 3B). These changes were generally transient, since dendritic spine size was not significantly different between before and after the contact (*p*>0.9; Figure 3C). No correlation between size change and putative contact duration was noted (*p*>0.9; Figure S8A), but the size change and initial spine size were significantly correlated (*r* = 0.28; *p*<0.001; Figure 3D), with the smallest spines undergoing the largest size changes during contact. Indeed, the comparison of small and large dendritic spines revealed a significant difference in their structural changes during putative microglial contact (average size differential for large spines: 1%±3%; for small spines: 17%±5%; *p*<0.01; Figures 3E, 3F, S8B, and S8C). Therefore, these observations indicate that microglial processes preferentially localize to small and structurally dynamic dendritic spines.

To determine whether spines targeted by microglia exhibit a different long-term fate with respect to their longevity, we carried out chronic in vivo imaging experiments tracking individual dendritic spines over a period of 2 d (Figure 3G and 3H). Surprisingly, we found that dendritic spines that received putative microglial contact during the first imaging session were more frequently eliminated (24%±6%; *n* = 30 spines in four animals) than non-contacted spines (7%±3%; *p*<0.05; *n* = 56 spines in four animals). Interestingly, among contacted dendritic spines, only small spines were lost (8/18 small spines and 0/12 large spines lost). We conclude that the subset of small and dynamic dendritic spines contacted by microglia also have an increased rate of elimination over a span of 2 d. Our two-photon imaging results raise the intriguing possibility that microglia may not only monitor the functional status of synapses, but also exert control on structural changes or spine elimination either through direct contact or indirect signaling that requires close microglia–synapse proximity.

### Ultrastructural Relationships between Microglia and Synapses during Alterations in Sensory Experience

**Figure 4:**
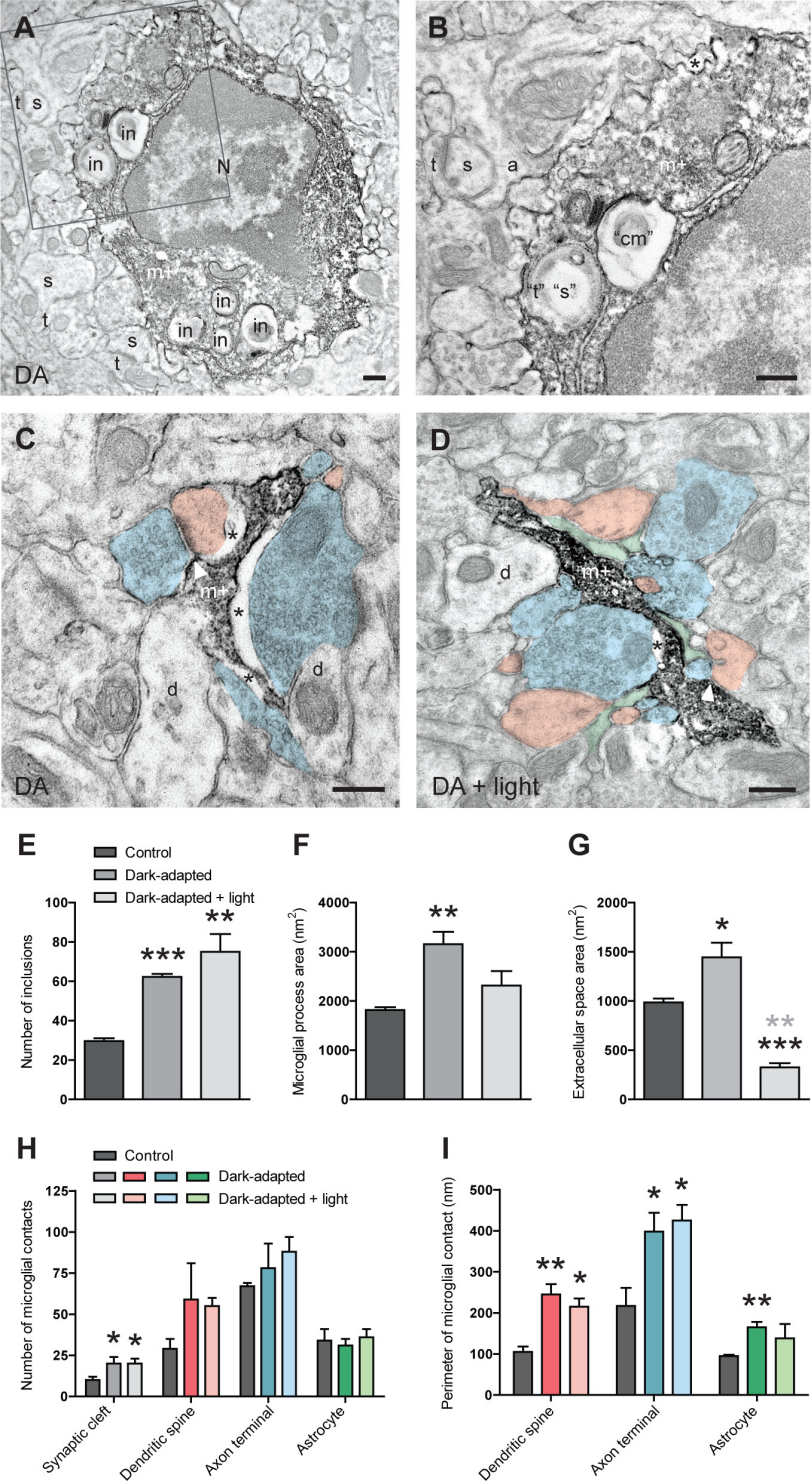
Ultrastructural relationships between microglia and synapses during altered visual experience. (A and B) EM image from a DA animal showing multiple cellular inclusions (in) in an IBA1-positive microglial perikaryon (m+). (B) shows a magnified view of the boxed region in (A). One inclusion resembles a dendritic spine (“s”) receiving a synapse from an axon terminal (“t”), while the other inclusion contains an accumulation of cellular membranes (“cm”). a, perisynaptic astrocyte; N, nucleus; s, dendritic spine; t, axon terminal. Scale bars = 250 nm. (C and D) EM images taken in DA (C) and DA+light (D) animals displaying two “spindly” microglial processes making multiple contacts with synapse-associated elements, including synaptic clefts (white arrowheads). Note that the process in (C) is surrounded by extended extracellular space (asterisks) in contrast with the process in (D). Color scheme as in Figure 1. d, dendrite. Scale bars = 250 nm. (E) Change in the total number of cellular inclusions for 50 IBA1-positive microglial processes during DA and DA+light (mean ± SEM). (F and G) Change in microglial process area and microglia-associated extracellular space area for 50 IBA1-positive microglial processes during DA and DA+light (mean ± SEM). (H and I) Total number of contacts and average perimeter of contact between 50 IBA1-positive microglial processes and every synapse-associated element (mean ± SEM). *, *p*<0.05; **, *p*<0.01; ***, *p*<0.001. Black asterisks refer to comparisons with control animals and grey asterisks to comparisons with DA animals. See also Figures S9 and S10 and Tables S1 and S2.

To investigate whether microglial behavior towards synapses is regulated by sensory experience, we altered visual experience by housing juvenile mice in complete darkness (dark adaptation [DA]) for 6 d, from the beginning to the peak of the critical period [12]. Binocular deprivation increases dendritic spine motility and turnover in mouse V1 in vivo [24],[25]; this provides an excellent model to correlate microglial behavior with the experience-dependent modification and elimination of synapses under non-pathological conditions. While microglial processes were generally larger in layer II of V1, they exhibited two morphological phenotypes at the ultrastructural level: some processes appeared very large and thick (“bulky”; Figure S10D and S10E), while others exhibited many short, thin fingers and appeared “spindly” (Figures 4C and S10F). Both types of microglial processes made multiple contacts with synapse-associated elements, including synaptic clefts (Figures 4C and S10D, S10E, S10F). Microglial perikarya and bulky processes often contained cellular inclusions (*p*<0.0001; *n* = 3 control and 3 DA animals; 50 IBA1-immunopositive microglial processes each; Figures 4A, 4B, 4E, S9A, S10D, and S10E), which sometimes resembled dendritic spines or axon terminals, suggesting their phagocytic engulfment by microglia. Spindly processes typically ensheathed dendritic spines or axon terminals and displayed extended extracellular space areas (Figures 4C and S10F). Microglial process area (*p*<0.01; *n* = 3 animals per condition; 50 IBA1-immunopositive processes each; Figure 4F; Table S1) and extracellular space area (*p*<0.05; *n* = 3 animals; Figure 4G; Table S1) were significantly increased during DA, with the correlation between microglial process area and extracellular space area remaining significant (*r* = 0.44; *p*<0.0001; *n* = 150 processes in three animals; Figure S9B). In contrast, microglial process density (DA: 131±6; control: 142±6 processes per 1,000 µm^2^ of neuropil; *n* = 3 animals; 1,000 µm^2^ of neuropil each; Figure S9C) and synaptic index (DA: 95%±0.6%; control: 94%±0.6%; *n* = 3 animals; Figure S9D) were unchanged by sensory experience (*p*>0.3 and *p*>0.1, respectively). Surprisingly, contacts with synaptic clefts were more frequent in DA animals (*p*<0.05; *n* = 3 animals per condition; total for 50 processes each; Figure 4H; Table S1) than controls, while contact frequency with dendritic spines, axon terminals, and astrocytic processes was unchanged (Figures 4H and S9E; Tables S1 and S2). Furthermore, as expected from extended microglial processes, their average perimeter of contact with every synapse-associated element was also increased during visual deprivation (spine: *p*<0.01; astrocyte: *p*<0.02; terminal: *p*<0.05; *n* = 3 animals per condition; 50 processes each; Figure 4I; Table S1). Thus, our ultrastructural observations reveal subtle experience-dependent changes in the behavior of microglia, most notably an expansion of their processes and associated extracellular space, an increased occurrence of cellular inclusions, an increased frequency of contact with synaptic clefts, and an increased apposition with every synapse-associated element.

To determine whether these changes in behavior could be reversed by reexposure to daylight, we subjected mice to DA for 6 d followed by a fixed 12-h light/dark cycle for 2 d (DA+light), as a few hours of reexposure to light after dark rearing (i.e., housing in complete darkness from birth) can cause rapid molecular changes at synapses, cause structural changes of dendritic spines, and reverse the effects of dark rearing on synaptic transmission and plasticity [26]–[29]. At the ultrastructural level in layer II of V1, most microglial process profiles appeared bulky during light reexposure, contained many inclusions, and were surrounded by small pockets of extracellular space (see Figures 4D and S10G, S10H, S10I for examples). Quantitative analysis revealed that microglial process area returned to control levels (*p*>0.2 versus control; *p*>0.09 versus DA; *n* = 3 animals per condition; Figure 4F) while microglia-associated extracellular space area was significantly reduced following light reexposure (*p*<0.004 versus control; *p*<0.02 versus DA; *n* = 3 animals per condition; Figure 4G). In contrast, the number of cellular inclusions, many of which contained elements resembling synaptic profiles (Figure S10G, S10H, S10I), remained significantly higher than in control animals (*p*<0.008 versus control; *p*>0.2 versus DA; *n* = 3 animals per condition; Figure 4E). Similarly, contacts with synaptic clefts (*p*<0.03 versus control; *p*>0.9 versus DA; *n* = 3 animals per condition; Figure 4H) and perimeters of contact with dendritic spines (*p*<0.01 versus control; *p*>0.4 versus DA; Figure 4I) and axon terminals (*p*<0.02 versus control; *p*>0.6 versus DA), but not with perisynaptic astrocytes (*p*>0.2 versus control and DA), remained extended. Future experiments with longer light exposures after deprivation will be needed to determine whether these phenomena can be reversed with further light reexposure. Taken together, these observations reveal a complex interaction between sensory-driven activity and microglial behavior. While the expansion of microglial processes and associated extracellular spaces reversed after brief reexposure to daylight, microglial ensheathment of dendritic spines and axon terminals, as well as their phagocytic inclusion, were still increased.

### Dynamic Relationships between Microglia and Synapses during Alterations in Sensory Experience

**Figure 5:**
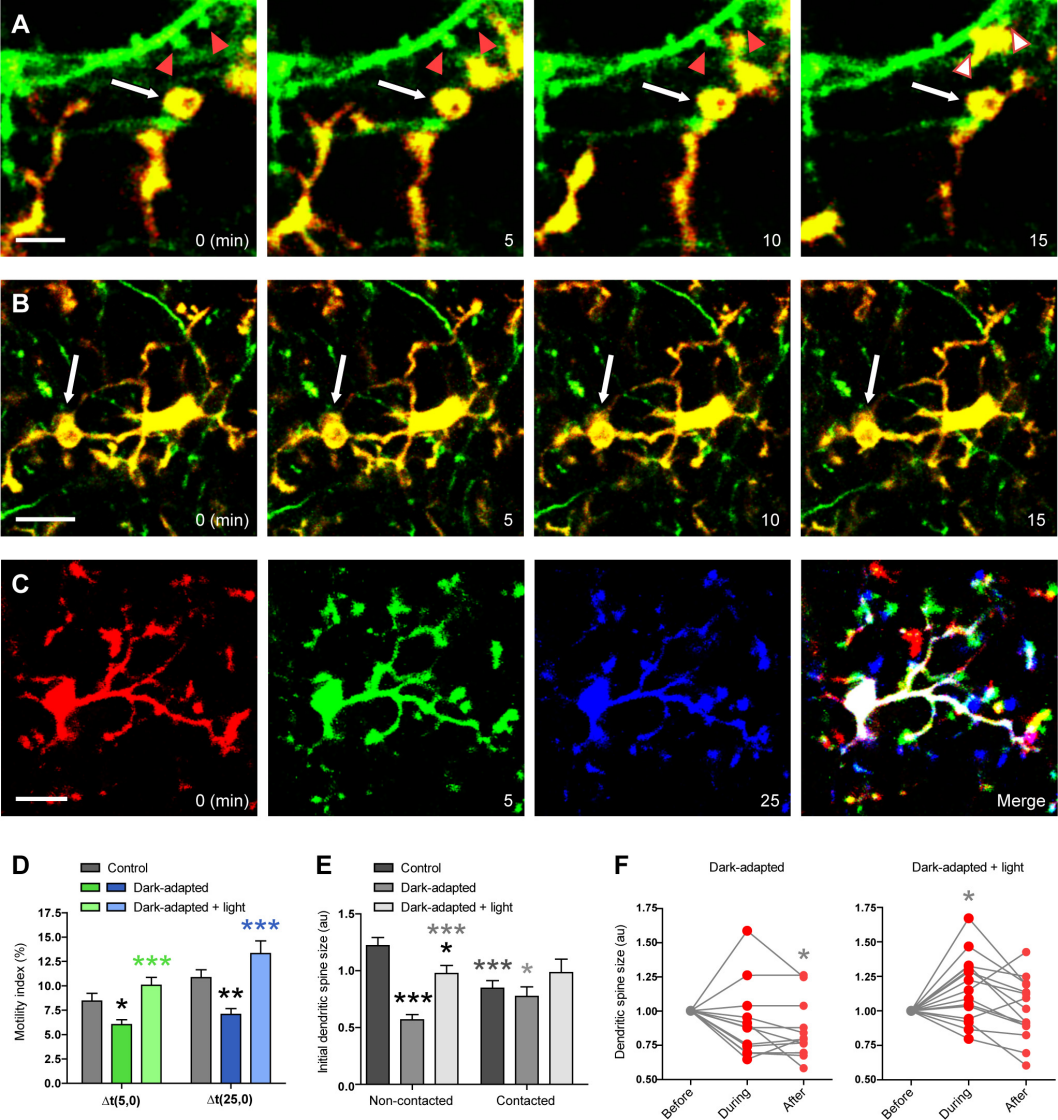
Changes in microglial behavior during altered visual experience. (A) Time-lapse images taken in a DA animal showing a microglial process that terminates into a structurally stable phagocytic cup (white arrow), while another process dynamically contacts two dendritic spines (red arrowheads before the contact; white arrowheads during the contact). Scale bar = 5 µm. (B) Time-lapse images showing another example of a structurally stable phagocytic cup (white arrow) in a DA animal. Scale bar = 10 µm. (C) Time-lapse images showing the motility of microglia during normal visual experience over three time points, 0, 5, and 25 min. Images from each time point were colored in red, green, and blue, respectively, and then merged to reveal microglia-associated pixels that were unchanged throughout the three time points (stable; white), changed in one of the three time points (dynamic; yellow and fuchsia), or changed in two of the three time points (highly dynamic; red, green, or blue). Scale bar = 10 µm. (D) Change in microglial motility index during DA and DA+light, measured as the proportion of the pixels that differed between images of a single microglia taken 5 or 25 min apart (mean±SEM). Black asterisks refer to comparisons with control animals, and green or blue asterisks to comparisons with DA animals. (E) Initial size of non-contacted and contacted dendritic spines in control, DA, and DA+light animals (mean ± SEM). Black asterisks refer to comparisons with non-contacted spines in control animals, dark grey asterisks to comparisons with non-contacted spines in DA animals, and light grey asterisks to comparisons with non-contacted spines in animals reexposed to light. (F) Dendritic spine size before, during, and after microglial contact in DA animals (left) and DA+light animals (right). Data were normalized to the first condition for presentation purposes. *, *p*<0.05; **, *p*<0.01; ***, *p*<0.001. See also Figures S11 and S12, as well as Videos S2, S3, S5, and S6.

To assess the dynamic changes in microglia–synapse interactions during visual deprivation, we used two-photon imaging of layers I/II of V1 in juvenile mice that were subjected to DA for 8–10 d, from the beginning to the peak of the critical period [12]. Microglial processes appeared thickened and sparse, and more often terminated into crown-like structures resembling phagocytic cups than in control animals [30] (Figure 5A and 5B; Video S2, as well as Figure S12 and Videos S4 and S5 for comparison of microglial morphology in control and DA animals). We also found that the average motility of microglial processes was significantly reduced (Figure 5C and 5D) when assayed in two ways: comparing morphology over a 5-min interval (motility index; control: 8%±0.8%; DA: 6%±0.5%; *p*<0.05; *n = *10 microglia in four control animals and 8 microglia in four DA animals) and over a 25-min interval, where the difference between control and DA animals was more pronounced (control: 11%±0.8%; DA: 7%±0.6%; *p*<0.01). Quantitative analysis of dendritic spines showed that spines not receiving putative microglial contact were significantly smaller in DA animals than in non-light-deprived animals (*p*<0.001; *n = *45 spines in three animals per experimental condition), whereas contacted spines were equally small in both DA and control animals (*p*>0.5; Figure 5E), in agreement with an observed reduction in synaptic strength during binocular deprivation [31],[32] and the smaller sizes of dendritic spines during synaptic depression [33],[34]. Surprisingly, dendritic spines putatively contacted by microglia in DA animals were significantly bigger than non-contacted spines (*p*<0.05), revealing that microglia no longer localize to smaller spines in this condition. The average duration of putative microglial contact with dendritic spines was slightly increased in DA animals (10±2 min; *n = *13 contacts in three animals) compared with controls (9±1 min; *n* = 37 contacts in five animals; Figure S11A), but the difference was not significant (*p*>0.8), suggesting that increased coverage of synaptic elements by microglia is not a result of longer duration of contact. Similarly, the frequency of putative contacts with individual dendritic spines was unchanged by sensory experience (DA: 1±0.08; control: 1±0.07 contacts per 40 min; *p*>0.6; Figure S11B). Lastly, most dendritic spines shrank during microglial contact (29% grew, 57% shrank, and 14% remained stable; *n* = 12 spines and 14 putative contacts in three animals), unlike in the control condition, where most spines grew. While average size changes during contact were not significant (size differential: −4%±4%; *p*>0.3; Figure S11C), interestingly, dendritic spine shrinkage persisted after microglial contact, with a significant difference in size between before and after the contact (*p*<0.02; Figure 5F), a phenomenon which may contribute to the reduction in size of the spine population. These results indicate that sensory-deprived microglia undergo subtle behavioral changes reminiscent of activation, including reduced motility, thickened processes, and phagocytic specializations. Intriguingly, despite unaltered duration and frequency of microglial contacts with synaptic elements, their preference of localization did change, from a subset of smaller dendritic spines that transiently grow to a subset of bigger spines that persistently shrink.

To further investigate these experience-dependent changes in microglial behavior, juvenile animals that were subjected to DA for 6–8 d were reexposed to daylight for 2 d before two-photon imaging of layers I/II of V1. Microglial morphologies resembled those in control animals, with generally thinner and more abundant processes within the neuropil (Figure S12; Video S6), but microglial processes still displayed phagocytic structures (Video S3). Microglial motility in animals reexposed to light was similar to that in control animals and was significantly increased compared with DA animals, when assessed during a 5-min interval (motility index = 10%±0.8%; *p*>0.2 versus control; *p*<0.001 versus DA; eight microglia in three DA+light animals; Figure 5D) and a 25-min interval (motility index = 13%±1.3%; *p*>0.1 versus control; *p*<0.0007 versus DA). Quantitative analysis of dendritic spines showed that spines receiving putative microglial contacts were of similar sizes to those in non-light-deprived and light-deprived animals (*p*>0.1 versus control and DA; *n* = 14 contacted spines in three DA+light animals; Figure 5E), while non-contacted spines were significantly bigger in animals reexposed to light (*p*<0.01 versus control; *p*<0.0001 versus DA; *n* = 45 spines in three animals per condition). The sizes of contacted and non-contacted spines were not significantly different in animals reexposed to light (*p*>0.9; Figure 5E), indicating that microglia no longer localize to specific spine types as in control or DA animals. Lastly, microglia–synapse interactions showed similar structural effects on dendritic spines as in control conditions. Most dendritic spines increased in size during putative microglial contact (67% grew, 20% shrank, and 13% remained stable; average size differential: 13%±5%; *p*<0.01 comparing with/without and before/during contact; *n* = 14 spines and 15 putative contacts in three animals; Figures 5F and S11C), and this growth was transient (*p*>0.8 comparing size differential before/after contact; Figure 5F). These results reveal additional changes in microglial behavior that can be reversed by brief reexposure to daylight, particularly their motility and preference of contact for subsets of dendritic spines.

## Discussion

Beyond immune surveillance, the physiological roles of quiescent microglia at synapses are unknown. Here we show that different modalities of microglial behavior are subtly altered by sensory experience, including regulation of extracellular spaces, apposition and phagocytosis of synaptic elements, dynamic interaction with subsets of dendritic spines, and motility of microglial processes.

### Microglial Regulation of Perisynaptic Extracellular Spaces

One of the most striking findings of our study was the demonstration of distinctive extracellular spaces closely correlated with the presence of microglial processes. Our EM observations revealed large electron-lucent pockets of extracellular space surrounding microglia. To our further surprise, we also observed changes in the distribution of these microglia-associated extracellular spaces during alterations in visual experience: an expansion during light deprivation and shrinkage during light reexposure. Although alteration of these spaces by brain fixation and embedding for EM may warrant further investigation, we observed them under all conditions tested. In future experiments, it will be important to determine whether microglial processes create this extracellular space themselves or move in to fill space that is created by an unknown mechanism. In any case, our findings of microglia-specific extracellular spaces suggest that microglia have an intimate relationship with their extracellular milieu and may even regulate their surrounding environment in a unique and specific way that is determined by physiological conditions.

If microglia directly modulate the extracellular space, they may do this through the secretion of various proteases that degrade extracellular matrix proteins, including cathepsins, metalloproteases, and tissue-type plasminogen activator [35]. This proteolytic degradation of specific matrix proteins could, in turn, facilitate microglial motility, as suggested by the finding that the migratory behavior of cathepsin S–deficient microglia is severely impaired in vitro [35]. In line with this, the volume of extracellular space greatly decreases during postnatal cortical development, concomitant with changes in extracellular matrix composition and reductions in cell migration and process elongation [14]–[16],[36],[37]. The appearance of extracellular spaces specifically associated with microglial processes could therefore reflect their highly motile behavior, relative to other structural elements of neuropil in juvenile cortex. Additionally, regulation of the extracellular matrix composition by microglia-derived proteases could contribute to dendritic spine motility and pruning, as well as activity-dependent and experience-dependent plasticity, which are profoundly affected in vitro and in vivo by treatments with proteases that degrade extracellular matrix proteins [38]–[43].

### Microglial Apposition and Phagocytosis of Synaptic Elements

Immunocytochemical EM and SSEM with 3-D reconstructions enabled us to analyze the morphology of microglial processes and their ultrastructural relationships with the other subcellular compartments of neuropil—astrocytic processes, axon terminals, and dendritic spines—in situ at high spatial resolution. Building on previous EM observations that microglia contact axon terminals and dendritic spines [9],[44],[45], our quantitative analysis revealed that most microglial processes directly appose not only axon terminals and dendritic spines, but also perisynaptic astrocytic processes and synaptic clefts. Our SSEM with 3-D reconstructions also uncovered the 3-D relationships between microglia and synapses, revealing that microglial processes contact multiple synapse-associated elements at multiple synapses simultaneously. Additionally, we found clathrin-coated pits at interfaces between microglia and dendritic spines, axon terminals, or perisynaptic astrocytic processes, suggesting clathrin-mediated endocytosis of membrane-bound receptors and their ligands, a phenomenon known to initiate various cellular signaling events [46]. Since clathrin-coated pits also occur at the tips of most spinules undergoing invagination, as previously observed in small dendritic spines, axon terminals, and perisynaptic astrocytic processes of mouse hippocampus [47], this may suggest trans-endocytosis of membrane-bound receptors and their ligands, in addition to a direct exchange of cytoplasm, between microglia and synapse-associated elements. To better understand the functional significance of these forms of molecular communication between microglia and synapse-associated elements, it will be important to identify the molecules that are being internalized during their dynamic interactions.

Following visual deprivation and reexposure to daylight, our EM results revealed that microglial processes change their morphology, appose synaptic clefts more frequently, and envelop synapse-associated elements more extensively. Since glial presence at the synaptic cleft was classically restricted to astrocytic processes regulating synaptic function through modification of the extracellular space geometry and bidirectional communication with synaptic elements [48],[49], this novel finding suggests that microglia may also contribute uniquely to synaptic transmission and plasticity in the healthy brain. Additionally, the ensheathment of synaptic elements and synaptic clefts by microglial processes may imply their participation in activity-dependent synapse elimination [50], through a separation of pre- and postsynaptic elements reminiscent of synaptic stripping, as previously reported between axon terminals and neuronal cell bodies during immune responses [8]. Lastly, our EM and two-photon in vivo imaging observations revealed that a subpopulation of microglial processes displays phagocytic structures, with an increasing prevalence during alterations in visual experience, thus providing evidence for a microglial role in phagocytic engulfment under non-pathological conditions. This is supported by observations that quiescent microglia can spontaneously engulf tissue components in vivo [10] and that activated microglia play an essential role in phagocytosis of cellular debris [1]–[3],[5],[6]. At the ultrastructural level, we also found cellular inclusions that resembled dendritic spines or axon terminals, suggesting that quiescent microglia can phagocytose synaptic elements in juvenile cortex. In line with this, classical complement proteins C1q and C3 were recently shown to be involved in the pruning of inappropriate retino-geniculate connections during early postnatal development [51]. Since C1q and downstream complement protein C3 can trigger a proteolytic cascade leading to microglial phagocytosis, this finding supports a model in which microglia may contribute to synaptic pruning under non-pathological conditions [52]. Taken together, our findings indicate that distinct modes of microglial interactions with synapses, most notably apposition, ensheathment, and phagocytosis, are subtly regulated by sensory experience.

### Dynamic Microglial Interaction with Subsets of Dendritic Spines

Two-photon visualization of microglia and synaptic elements with two different colors in CX_3_CR1-GFP/Thy1-YFP mice enabled clear distinction of the separate structures, which facilitated identification of their putative contacts. This approach revealed transient localization of microglial processes to the vicinity of dendritic spines and axon terminals, in agreement with observations from a recent study [9]. In our study, clear distinction of the separate structures also allowed, for the first time, measurement of dendritic spine and axon terminal morphological changes during episodes of proximity with microglial processes. Our results revealed a specificity of these putative microglial contacts for a subset of small, transiently growing, and frequently eliminated dendritic spines. This is consistent with previous findings showing that in mouse visual and somatosensory cortical areas in vivo, small dendritic spines are more motile and more frequently eliminated than their larger counterparts [20],[22],[24],[53],[54]. Dendritic spines undergoing long-term potentiation transiently or persistently enlarge in vitro [33],[34],[55],[56], but since dendritic spine structural changes can occur independently of changes in synaptic strength [57], a connection between putative microglial contact and synaptic plasticity remains to be determined. During light deprivation, microglia preferentially localized to larger dendritic spines that persistently shrank, akin to spines undergoing long-term depression in vitro [33],[34] or shrinking before elimination in vivo [58], while during reexposure, microglia reversed to contacting spines that transiently grew, as in control animals. This uncovers a specificity of microglial interaction for subsets of structurally dynamic and transient dendritic spines, a specificity that, surprisingly, changes with sensory experience.

In future experiments, correlating the temporal dynamics of microglial contact with dendritic spine activity could be achieved by in vivo labeling of neurons with electroporated calcium indicators [59] or viral delivery of genetic calcium indicators [60]. A challenge with such techniques will be their invasive nature; the mechanical disruption of the brain alone during indicator delivery can result in microglial activation. Additionally, it will be important to determine whether microglial contacts occur in response to structural changes in dendritic spines and whether such contacts instruct subsequent spine elimination. This will, however, require new technical advances enabling interference with microglial contacts while preserving physiological conditions. Importantly, whether direct microglial contacts, such as observed with EM, are required for these structural changes or whether microglia can exert effects on synapses without close apposition will need to be explored.

### Motility of Microglial Processes

Several lines of evidence, including those presented here, suggest that microglial motility may be regulated by neuronal activity. A pioneer study reported an increase in the volume sampled by quiescent microglia in vivo over a period of 1 h after application of the ionotropic GABA receptor blocker bicuculline, whereas the sodium channel blocker tetrodotoxin had no significant effects [10]. More recently, microglia were shown to retract their processes and reduce their frequency of contact with axon terminals in vivo, over a period of 4–6 h after binocular enucleation or intraocular injection of tetrodotoxin [9]. In our study, two-photon analysis revealed a global decrease in microglial motility during light deprivation without correlated changes in the duration or frequency of microglial contact with individual dendritic spines. This may highlight differences between short-term (1–6 h) and long-term (8–10 d) microglial responses to sensory deprivation or distinguish between more invasive manipulations, which approximate nervous system injury, and more physiological paradigms such as DA. Our observations further suggest that subsets of microglial processes may have different behavior: those in contact with dendritic spines may be highly motile, while others (for example bulky microglial processes displaying phagocytic structures) may become less motile. It is also possible that spindly microglial processes (<100 nm), which were typically devoid of cellular inclusions and surrounded by extended extracellular space, were undetected with two-photon imaging and therefore not accounted for in our motility analysis. This could explain the finding that microglial motility decreased during light deprivation while microglia-associated extracellular spaces expanded. However, as we found an increase in microglial motility during light reexposure that was accompanied by a decrease in microglia-associated extracellular space areas, it is likely that microglial motility and extracellular space areas are regulated independently.

### Conclusion

Using several technical approaches, we were able to provide a qualitative and quantitative characterization of the ultrastructural and structural/dynamic interactions between microglia and synapses under non-pathological conditions. This characterization revealed specific modalities of microglia–synapse interactions that are subtly altered by sensory experience, supporting the exciting possibility that microglial influence on synaptic plasticity is not restricted physiologically to an immune response to brain injury and disease.

## Materials and Methods

### Animals

Animals were treated in strict accordance with the University of Rochester Committee on Animal Resources and the United States National Institutes of Health standards. Light-reared animals were housed under a fixed 12-h light/dark cycle; DA animals were placed in complete darkness for 6–10 d, from P20–P22 until P28–P32 (the onset of experimentation); DA+light animals were housed under a fixed 12-h light/dark cycle for 2 d following DA until P29–P32 (the onset of experimentation). For immunocytochemical EM and SSEM, eight C57Bl/6 mice (P28) were anesthetized with sodium pentobarbital (80 mg/kg, i.p.) and perfused through the aortic arch with 3.5% acrolein followed by 4% paraformaldehyde as previously described [14],[61] or with 2.75% glutaraldehyde in 2% paraformaldehyde to compare extracellular space areas surrounding microglia between both types of fixatives. For two-photon imaging [17], 21 CX_3_CR1-GFP/Thy1-YFP mice (P28–P39), in which microglia and cortical layer V neurons are respectively GFP- and YFP-labeled [17],[18], were anesthetized with a mixture of fentanyl (0.05 mg/kg, i.p.), midazolam (5.0 mg/kg), and metatomadin (0.5 mg/kg) [62] and kept at 37°C with a heating pad. Stereotaxical coordinates encompassing a 2- to 3-mm-diameter region (A +0.16 to A +0.64, between 2 and 3 mm from the midline) [63] were used to identify V1. In all cases, DA animals were anesthetized in the dark using infrared goggles or a darkroom red light before undergoing perfusion or surgery in the light. After acute (30 min to 2 h) and chronic (two imaging sessions over 2 d, 30 min to 2 h each) two-photon imaging, eight mice were perfused with 4% paraformaldehyde to confirm that microglia are not activated by the surgical procedure and imaging paradigm (Figure S4).

### Immunoperoxidase Staining for Light Microscopy and EM

Transverse sections of the brain (50 µm thick) were cut in ice-cooled PBS (0.9% NaCl in 50 mM phosphate buffer [pH 7.4]) with a vibratome. Sections were immersed in 0.1% sodium borohydride for 30 min at room temperature (RT), washed in PBS, and processed freely floating following a pre-embedding immunoperoxidase protocol previously described [14],[61],[64],[65].

Briefly, sections were rinsed in PBS, followed by a 2-h pre-incubation at RT in a blocking solution of PBS containing 5% normal goat serum and 0.5% gelatin. They were incubated for 48 h at RT in rabbit anti-IBA1 antibody (1∶1,000 in blocking solution; Wako Pure Chemical Industries) and rinsed in PBS. After incubation for 2 h at RT in goat anti-rabbit IgGs conjugated to biotin (Jackson Immunoresearch) and with streptavidin-horseradish peroxidase (Jackson Immunoresearch) for 1 h at RT in blocking solution, labeling was revealed with diaminobenzidine (0.05 mg/ml) and hydrogen peroxide (0.03%) in buffer solution (DAB Peroxidase Substrate Kit; Vector Laboratories).

Sections for light microscopy were mounted onto microscope slides, dehydrated in ascending concentrations of ethanol, cleared in xylene, and coverslipped with DPX (Electron Microscopy Sciences). Sections for EM were post-fixed flat in 1% osmium tetroxide and dehydrated in ascending concentrations of ethanol. They were treated with propylene oxide, impregnated in Durcupan (Electron Microscopy Sciences) overnight at RT, mounted between ACLAR embedding films (Electron Microscopy Sciences), and cured at 55°C for 48 h. Areas of V1, at a level approximating the transverse planes A +0.16 to A +0.72 [63], were excised from the embedding films and re-embedded at the tip of resin blocks. Ultrathin (65–80 nm) sections were cut with an ultramicrotome (Reichert Ultracut E), collected on bare square-mesh grids, stained with lead citrate, and examined with a Hitachi 7650 electron microscope.

### Light Microscopy Imaging

Light microscope pictures of IBA1 immunostaining were taken at 20× in layer II of V1, using a Spot RT color digital camera (Diagnostic Instruments).

### EM Imaging and Data Analysis

Eighty pictures were randomly taken at 40,000× in layer II of V1 in each animal (*n* = 3 control, 3 DA, and 3 DA+light), corresponding to a total surface of ∼1,000 µm^2^ of neuropil per animal. Cellular profiles were identified according to criteria previously defined [14],[44],[66],[67]. In addition to their IBA1 immunoreactivity, microglial cell bodies were recognized by their small size and the clumps of chromatin beneath their nuclear envelope and throughout their nucleoplasm. Microglial processes displayed irregular contours with obtuse angles, dense cytoplasm, numerous large vesicles, occasional multivesicular bodies, vacuoles or cellular inclusions (large lipidic vesicles, profiles of cellular membranes, and profiles of other structural elements including dendritic spines and axon terminals), and distinctive long stretches of endoplasmic reticulum. These morphological characteristics enabled identification of microglial processes on an ultrastructural level in non-immunocytochemical SSEM and non-immunocytochemical glutaraldehyde-fixed material.

In each of three control and three DA animals (∼1,000 µm^2^ of neuropil per animal), IBA1-immunopositive microglial process profiles were counted and their contacting structural elements identified. A synaptic index was calculated based on the percentage of IBA1-positive microglial processes contacting synapse-associated elements divided by the total number of IBA1-positive microglial processes (Figure S9). It is important to note that since analysis was performed on single sections, different microglial process profiles could be part of a single microglial process, which may lead to an overestimation of the synaptic index in this analysis. In 39 pictures in each of three control animals (∼500 µm^2^ of neuropil per animal), all extracellular space areas wider than synaptic clefts (10–20 nm) [66] were measured and determined to contact or not contact IBA1-stained microglial processes (Figure 1; Table S3).

To characterize the ultrastructural relationships between microglia and synapse-associated elements, 50 randomly selected IBA1-positive microglial processes per animal were analyzed in more detail with Image J software (United States National Institutes of Health). For measurement of microglial process and extracellular space areas, individual microglial processes and all their adjacent extracellular spaces wider than synaptic clefts were traced with the freehand line tool. Their area was quantified in pixels and converted into nanometers (Figures 1, 4, and S9; Table S1). For quantification of contacts between microglial processes and synapse-associated elements, we first counted all direct juxtapositions between individual microglial processes and dendritic spines, axon terminals, synaptic clefts, and perisynaptic astrocytic processes (Figure 4; Table S1). For example, microglial processes contacting synaptic clefts were also contacting dendritic spines and axon terminals. Since most microglial processes contacted multiple synapse-associated elements simultaneously, we performed a second analysis where we categorized individual microglial processes based on their contacting partners: dendritic spine only, axon terminal only, perisynaptic astrocytic process only, spine+terminal, spine+astrocytic process, terminal+astrocytic process, or spine+astrocytic process+terminal. Microglial contacts with synaptic clefts were included in the spine+terminal or spine+astrocytic process+terminal categories (Table S2). For measurement of perimeters of contact between microglia and dendritic spines, axon terminals, or perisynaptic astrocytic processes, all microglial cell membranes in direct juxtaposition with these structural elements were traced with the freehand line tool (Figure 4; Table S1). Their length was quantified in pixels and converted into nanometers.

### SSEM, 3-D Reconstructions, and Quantitative Analysis

A series of 100 ultrathin sections (65 nm) was cut, collected on pioloform-coated grids, stained with lead citrate, and examined with a JEOL JEM-1230 electron microscope. Fifty serial images of a microglial process were taken at 10,000×. The alignment of images, tracing of structural elements, and 3-D reconstructions were performed with the Reconstruct software [68]. To calculate the volume of 15 extracellular spaces, we measured their area in all sections in which they appeared and multiplied their total area by the number and thickness of sections.

### Two-Photon In Vivo Imaging and Data Analysis

The skull above V1 was exposed, cleaned, glued to a thin metal plate, and carefully thinned to an approximately 20- to 30-µm thickness, using a high-speed dental drill (Fine Science Tools) and a microsurgical blade [19],[24],[69]. Drilling was interrupted periodically, and sterile saline was applied on the skull to prevent heat-induced damage.

A custom-made two-photon microscope [70] with a Ti∶Sapphire laser (Mai Tai; Spectra Physics) tuned to 920 nm was used for transcranial imaging. Fluorescence was detected using two photomultiplier tubes in whole-field detection mode and a 506-nm dichroic mirror with 580/180 (green channel: GFP) and 534/34 (yellow channel: GFP and YFP) emission filters. A 20× water-immersion lens (0.95 N.A.; Olympus) was used throughout the imaging session. Dendrites and axons near microglia, located at least 50 µm below the pial surface, were imaged under a digital zoom of 4–6×, using the FluoView software. *Z* stacks taken 1 µm apart were acquired every 5 min for 30 min to 2 h. To measure the resolution of two-photon imaging under our experimental conditions, we imaged subresolution fluorescent beads (0.1–0.2 µm diameter; Molecular Probes) to obtain the experimental point spread function. The measured 1/*e* radius was 0.35 µm radially and 1.3 µm axially, corresponding to the *X*/*Y* and *Z* resolution, respectively.

Analysis of microglial contacts with dendritic spines (*n* = 5 control animals, 3 DA animals, and 3 DA+light animals) and axon terminals (*n* = 3 control animals), changes in synaptic structures (*n* = 3 and 5 control animals with terminals and spines, respectively, 3 DA animals with spines, and 3 DA+light animals with spines), dendritic spine turnover (*n* = 4 control animals), and microglial motility (*n* = 4 control animals, 4 DA animals, and 3 DA+light animals) was performed with Image J software. Axon terminals were identified as finger-like protrusions (>0.2 µm) from the axon or bead-like structures along the axon (at least two times the axon diameter), as previously described [54]. While dendritic spine morphology exists as a continuum, different behaviors have been assigned to spines with different structures [71]. Therefore we classified dendritic spines into mushroom, thin, and stubby types based on their length and spine head volume, as previously described [24]. The only three stubby spines contacted by microglia were excluded from the analysis of spine size because of their rarity and the difficulty of measuring their fluorescence intensity. Consequently, we also removed stubby spines from the analysis of size for non-contacted and contacted dendritic spines. Among thin and mushroom spines, we determined a range of normalized dendritic spine sizes. The spines belonging to the first quarter of this range were considered “small,” and those in the other three-quarters were identified as “large.” Filopodia were rarely observed at these ages and were excluded from the analysis.

For visualization of microglial contacts with dendritic spines and axon terminals, the green channel was arbitrarily assigned the color red and the yellow channel assigned the color green, enabling the visualization of microglia in yellow and neuronal elements in green (see Figure S6). Microglial contacts were identified manually by stepping through the *Z* stack without projection. All microglial contacts (colocalization of fluorescence for microglia and synaptic elements) that started and ended during imaging were included in the analysis.

For analysis of changes in synaptic structures, the background was subtracted from each of the two channels and the green channel bleedthrough was subtracted from the yellow channel (see Figure S6 for uncorrected and corrected images). In the stacks unadjusted for brightness or contrast, dendritic spines and axon terminals were analyzed for fluorescence at the *Z* level where they appeared brightest. A line was traced through each element, and a fluorescence plot profile was created, which was then fitted to a Gaussian. Because the majority of dendritic spines are below the resolution of our two-photon microscope [72], the maximal fluorescence (amplitude of the Gaussian fit) was used to assess the relative spine size (see Figure S8C for sizes of large spines assessed with the width of the fluorescence profile [1/*e* ^1/2^ radius of the Gaussian fit] to rule out underestimation of their size changes during putative contact). Since axon terminals are generally much larger than dendritic spines, the width of the Gaussian fit to the fluorescence profile was used to determine their relative size. To rule out contamination of neuronal measurements by any unsubtracted bleedthrough of microglial fluorescence, we analyzed thin fluorescent axons (*n* = 1 axon in each of 14 animals) that were contacted by microglia, using the height of the Gaussian fit to assess relative size. Axon size measured in this manner was relatively unchanged between populations with and without microglial contact (average size differential of −0.1%±1%; see Figure S6D and S6E), as expected for these structurally stable elements.

Size differentials of terminals and spines were calculated as the ratio of size difference with and without contact over the size without contact. In order to compare spine size between animals, we normalized spine fluorescence by the maximal fluorescence in the adjacent dendrite. Axon terminals and dendritic spines with size differentials under 1% were considered stable. For presentation purposes, we normalized the size of terminals and spines by dividing each individual structure's average size in the presence of microglial contact (with or during) by its average size in the absence of microglial contact (without, or before or after). We also normalized the size of terminals and spines by dividing each individual structure's average size by the size of the largest terminal or spine.

To determine the turnover of dendritic spines that were or were not contacted by microglia in the first imaging session, the position of individual dendritic spines was compared between time points separated by 2 d. The proportion of eliminated dendritic spines was defined as the proportion of spines from the original population not observed on the second day of imaging. Spines located more than 0.8 µm laterally from their previous location were considered to be new spines.

For measurement of microglial motility, images centered on the cell body from five consecutive *Z* levels were projected into two dimensions, for each microglia and each time point analyzed (0, 5, and 25 min). For each microglia, the images were aligned and grouped into a stack. Stacks were adjusted for brightness and contrast, and then binarized. For each microglia, the difference between images taken at the 0- and 5- or 25-min time points was calculated. A motility index was determined, as the proportion of the pixels that differed between the two images.

### Statistical Analysis

Analyses were performed with Prism 5 software (GraphPad Software). All values reported in the text are mean ± standard error of the mean (SEM). For all statistical tests, significance was set to *p*<0.05. Two-tailed unpaired Student's *t* tests and linear regressions were used for both EM and two-photon analyses. For two-photon analyses, two-tailed paired Student's *t* tests were also used to compare the size of the same dendritic spines or axon terminals in the presence versus in the absence of microglial contact. Sample size (*n*) represents individual animals for EM (except for correlation analysis, where *n* represents individual extracellular space and microglial process areas) and synaptic elements or microglial contacts for two-photon analyses (except for analysis of dendritic spine turnover, where *n* represents individual animals).

## Supporting Information

Figure S1 **Light microscopic image showing immunoperoxidase staining for IBA1, under the same immunocytochemical conditions as used for EM.** The staining is restricted to microglia, which shows its specificity. Scale bar = 50 µm.
(0.88 MB TIF)

Figure S2 **Other views of the 3-D reconstruction that further reveal the geometry of the microglia-associated extracellular spaces.** In (A), axon terminals (blue), dendritic spines (red), and perisynaptic astrocytes (green) are made semitransparent. Taupe indicates microglia. In (B), the axon terminals are removed from the display, while in (C) only the microglial process and extracellular space are shown. Scale bars = 250 nm.
(1.45 MB TIF)

Figure S3 **SSEM images showing additional examples of coated pits at the sites of cell–cell contact between microglia and synapse-associated elements.** In these examples, the vesicles that appear coated (black arrows) are found inside a microglial process (m), at the sites of contact with an astrocytic process (a) (A), a dendritic spine (s) (B), and an axon terminal (t) (C). d, dendrite; N, nucleus; p, perikaryon. Scale bar = 250 nm.
(2.05 MB TIF)

Figure S4 **SSEM images from an animal undergoing normal visual experience, showing the phagocytic engulfment of cellular debris by a microglial process.** Images are separated by 65 nm. *, extracellular space; a, astrocyte; d, dendrite; in, cellular inclusion; m, microglial process; N, nucleus; p, perikaryon; s, dendritic spine; t, axon terminal. Scale bars = 250 nm.
(4.02 MB TIF)

Figure S5 ***Z*** **projections showing the morphology of microglia in brain sections of animals perfused after two-photon in vivo imaging.** The imaged area is shown in (A), and the corresponding contralateral area is shown in (B). The pial surface is presented at the top of the image in both cases. The similar polarity, thickness, and density of microglial cell bodies and processes (green) confirm that microglia are not activated by transcranial imaging. Scale bar = 15 µm.
(1.47 MB TIF)

Figure S6 **Separation of GFP and YFP fluorescence in CX_3_CR1-GFP/Thy1-YFP mice. (**A) Two-photon images from the yellow channel (YFP+GFP; left) and green channel (GFP; right) in their uncorrected state. (B) Two-photon image from the yellow channel corrected with subtraction of background and GFP fluorescence for analysis of axon terminal and dendritic spine sizes. (C) Merge of the yellow and green channels (assigned the colors green and red, respectively) and adjustment of brightness and contrast for visualization of microglial contacts (yellow) with neuronal elements (green). (D) Two-photon image showing a contact between a microglial process (yellow) and a small axon (green; white arrowhead) during normal sensory experience. (E) Axon size without versus with microglia contact, normalized to the first condition for presentation purposes. au, arbitrary units.
(1.42 MB TIF)

Figure S7 **Structural/dynamic interactions between microglia and axon terminals during normal visual experience in vivo.** (A) Time-lapse image showing an axon terminal (green; blue arrowhead) contacted by microglial processes with bulbous endings (yellow; white arrowhead) over 20 min. Scale bar = 5 µm. (B and C) Axon terminal size without versus with microglial contact (B) or before, during, and after contact (C), normalized to the first condition for presentation purposes. (D and E) Lack of correlation between microglial contact duration or initial terminal size (normalized to largest axon terminal) and the change in axon terminal size during microglial contact. au, arbitrary units.
(1.17 MB TIF)

Figure S8 **Additional analysis of structural/dynamic interactions between microglia and dendritic spines during normal visual experience in vivo.** (A) Lack of correlation between microglial contact duration and the change in dendritic spine size during contact. (B and C) Change in the size of large dendritic spines assessed with the amplitude of the Gaussian fit to the fluorescent profile (as for other analyses of dendritic spine size presented in the Results section) or with the width of the fluorescent profile (1/*e* ^1/2^ radius of the Gaussian fit; see Materials and Methods section), confirming that the size changes of bigger spines were not underestimated with assessments of maximal fluorescence.
(0.13 MB TIF)

Figure S9 **Additional analysis of ultrastructural interactions between microglia and synapse-associated elements during altered visual experience.** (A) EM image taken in a DA animal showing a microglial (m+) perikarya that contains vacuole (vac) and cellular inclusions (in). *, extracellular space; ma, myelinated axon; N, nuleus. Scale bar = 250 nm. (B) Correlation between the areas of microglial processes and associated extracellular space in DA animals. (C) Total number of IBA1-immunopositive microglial processes in a surface of 1,000 µm^2^ of neuropil, in control versus DA animals (*n* = 3 animals per experimental condition; mean ± SEM). (D) Synaptic index in control versus DA animals (*n* = 3 animals per experimental condition; mean ± SEM). (E) Proportion of simultaneous microglial contacts with one, two, or three synapse-associated elements (*n* = 3 control and 3 DA animals; mean ± SEM).
(1.17 MB TIF)

Figure S10 **EM images showing additional examples of microglial processes contacting multiple synapse-associated elements, including synaptic clefts, in the different experimental conditions.** a, perisynaptic astrocytic process; d, dendrite; m, microglial process, s, dendritic spine; t, axon terminal. White arrowheads indicate synaptic clefts. Scale bars = 250 nm. In (A–C; control animals), the small microglial processes (m+) are devoid of cellular inclusions and surrounded by narrow extracellular space. In (D–F; DA animals), larger microglial processes (m+) display bulky (D and E) or spindly (F) morphologies. While the bulky processes contain cellular inclusions resembling profiles of dendritic spine (“s”), axon terminal (“t”), or cellular membranes (“cm”), the spindly process is surrounded by extended extracellular space. In (G–I; DA+light animals), the large-to-small microglial processes display vacuole (vac) and cellular inclusions resembling terminals (“t”) as well as a synapse between a dendritic spine (“s”) and a terminal (“t”). Little extracellular space is observed.
(7.32 MB TIF)

Figure S11 **Additional analysis of structural/dynamic interactions between microglia and dendritic spines during altered visual experience in vivo.** (A and B) Duration and frequency of microglial contacts with individual dendritic spines during 40-min imaging sessions, in DA and control animals (mean ± SEM). (C) Dendritic spine size without versus with microglial contact in DA animals (left) and DA+light animals (right), normalized to the first condition for presentation purposes. *, *p*<0.05.
(0.14 MB TIF)

Figure S12 **Time-lapse images showing additional examples of microglial morphology and motility in the different experimental conditions.** Images from control (A), DA (B), and DA+light (C) animals are shown. In (B), note the thickening of microglial processes, which also are sparse. Scale bars = 10 µm. See also Videos S4–S6.
(6.14 MB TIF)

Table S1 **Changes in the ultrastructural interactions between microglia and synapse-associated elements with visual experience.**
(0.21 MB TIF)

Table S2 **Changes in the ultrastructural interactions between microglia and different combinations of synapse-associated elements with visual experience.**
(0.14 MB TIF)

Table S3 **Diversity in microglia-associated extracellular space volumes.**
(0.16 MB TIF)

Video S1 **Time-lapse video showing the structural/dynamic interactions between microglia and dendritic spines during normal visual experience in vivo.** Multiple contacts between microglial processes displaying bulbous endings (yellow) and dendritic spines (green), as well as contacts with dendritic branches (green), are observed over 1 h. Images were acquired at the same depth, approximately 50 µm below the pial surface.
(7.75 MB AVI)

Video S2 ***Z*** **stack showing examples of microglial processes ending in phagocytic cups during DA in vivo.** Images of microglia (yellow) were acquired 1 µm apart, starting approximately 50 µm below the pial surface. Two phagocytic cups are shown (arrows), with the smallest one surrounding a YFP-positive neuronal element (green).
(0.77 MB AVI)

Video S3 ***Z*** **stack showing examples of microglial processes ending in phagocytic cups in a DA+light animal in vivo.** Images were acquired 1 µm apart, starting approximately 50 µm below the pial surface. Three phagocytic cups are shown (arrows), with the small one at the left surrounding a YFP-positive neuronal element (green).
(0.97 MB AVI)

Video S4 **Time-lapse video showing the morphology and motility of microglia during normal visual experience in vivo.** Five microglia (yellow) contacting multiple axons (green) and axon terminals (green) are shown. Note the presence of a small structure resembling a phagocytic cup (arrow) near YFP-positive axonal elements (green). Images were acquired at the same depth, approximately 50 µm below the pial surface, every 5 min for 30 min.
(0.32 MB AVI)

Video S5 **Time-lapse video showing the morphology and motility of microglia during DA in vivo.** Four microglia (yellow) displaying sparse processes in the neuropil are shown. Images were acquired at the same depth, approximately 50 µm below the pial surface, every 5 min for 30 min.
(0.48 MB AVI)

Video S6 **Time-lapse video showing the morphology and motility of microglia during DA followed by reexposure to light in vivo.** Three microglia (yellow) contacting multiple dendrites (green) and dendritic spines (green) are shown. Images were acquired at the same depth, approximately 50 µm below the pial surface, every 5 min for 30 min.
(0.76 MB AVI)

## Abbreviations

3-D: three-dimensional
DA: dark adaptation
EM: electron microscopy
P: postnatal day
RT: room temperature
SEM: standard error of the mean
SSEM: serial section electron microscopy
V1: primary visual cortex
