# Supplementary figures and images for: Microglial Interactions with Synapses Are Modulated by Visual Experience
Source: PLoS Biol. 2010 Nov 2;8(11):e1000527. doi: 10.1371/journal.pbio.1000527 (PMC2970556; doi:10.1371/journal.pbio.1000527)

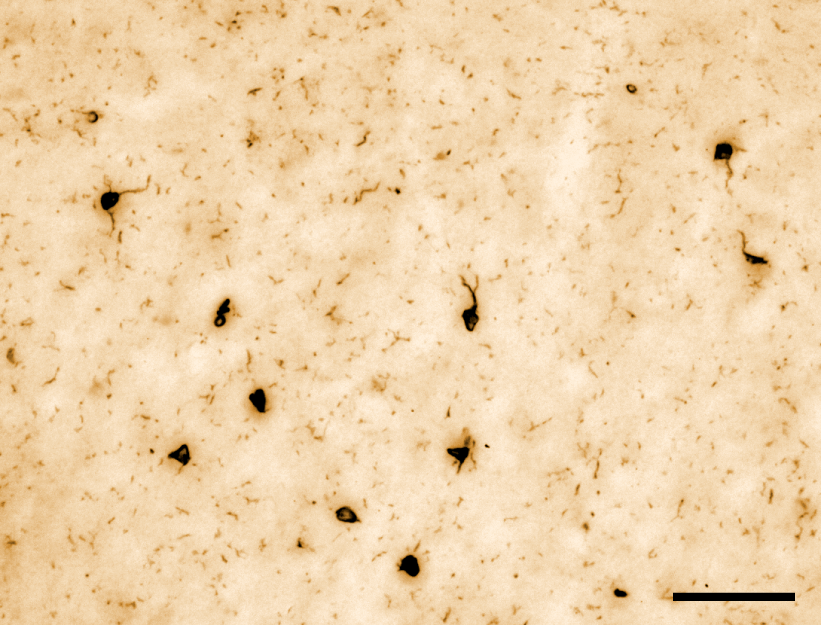

Supplement: Figure S1 — Light microscopic image showing immunoperoxidase staining for IBA1, under the same immunocytochemical conditions as used for EM. The staining is restricted to microglia, which shows its specificity. Scale bar = 50 µm. (0.88 MB TIF) [file pbio.1000527.s001.tif]

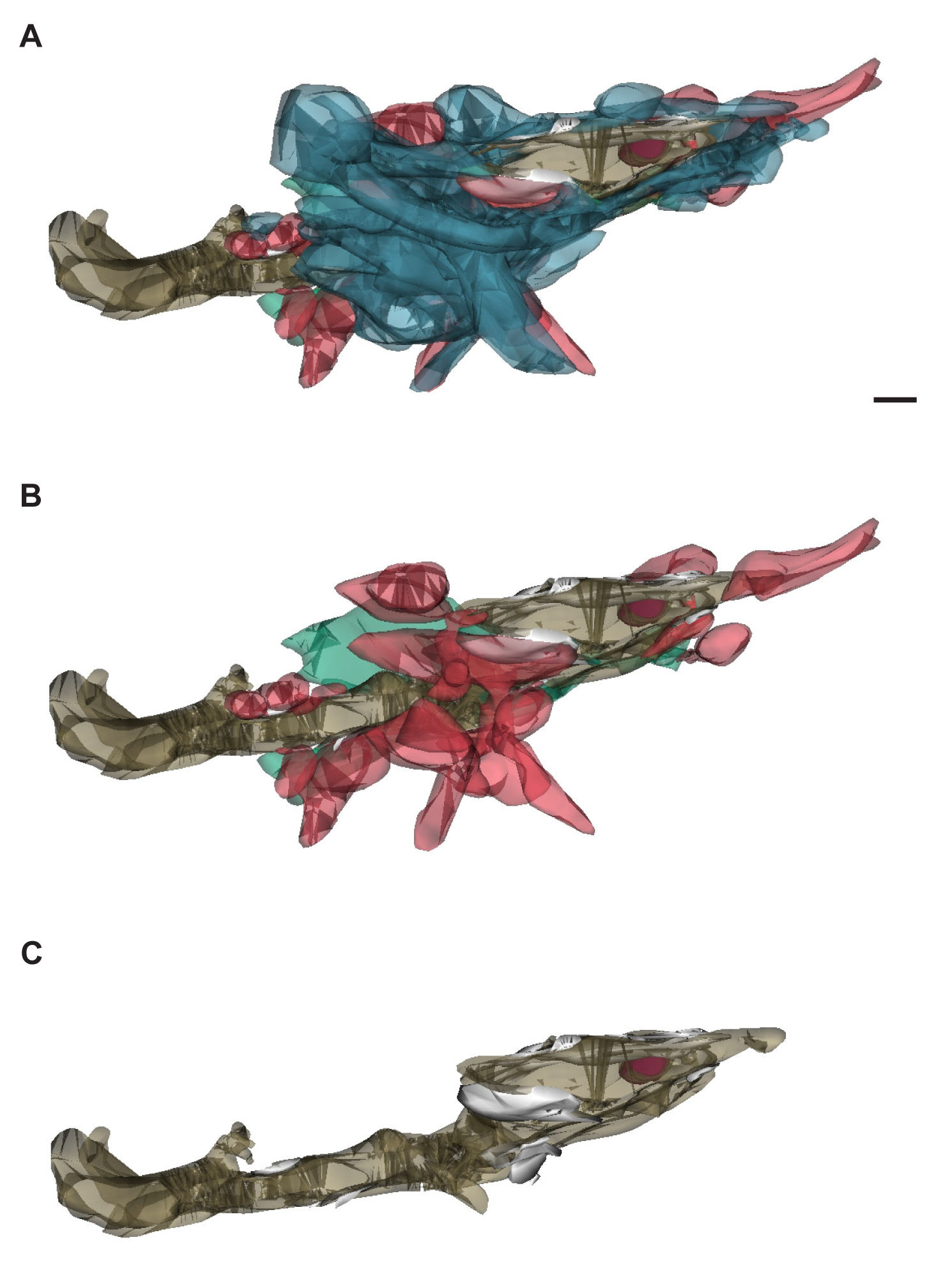

Supplement: Figure S2 — Other views of the 3-D reconstruction that further reveal the geometry of the microglia-associated extracellular spaces. In (A), axon terminals (blue), dendritic spines (red), and perisynaptic astrocytes (green) are made semitransparent. Taupe indicates microglia. In (B), the axon terminals are removed from the display, while in (C) only the microglial process and extracellular space are shown. Scale bars = 250 nm. (1.45 MB TIF) [file pbio.1000527.s002.tif]

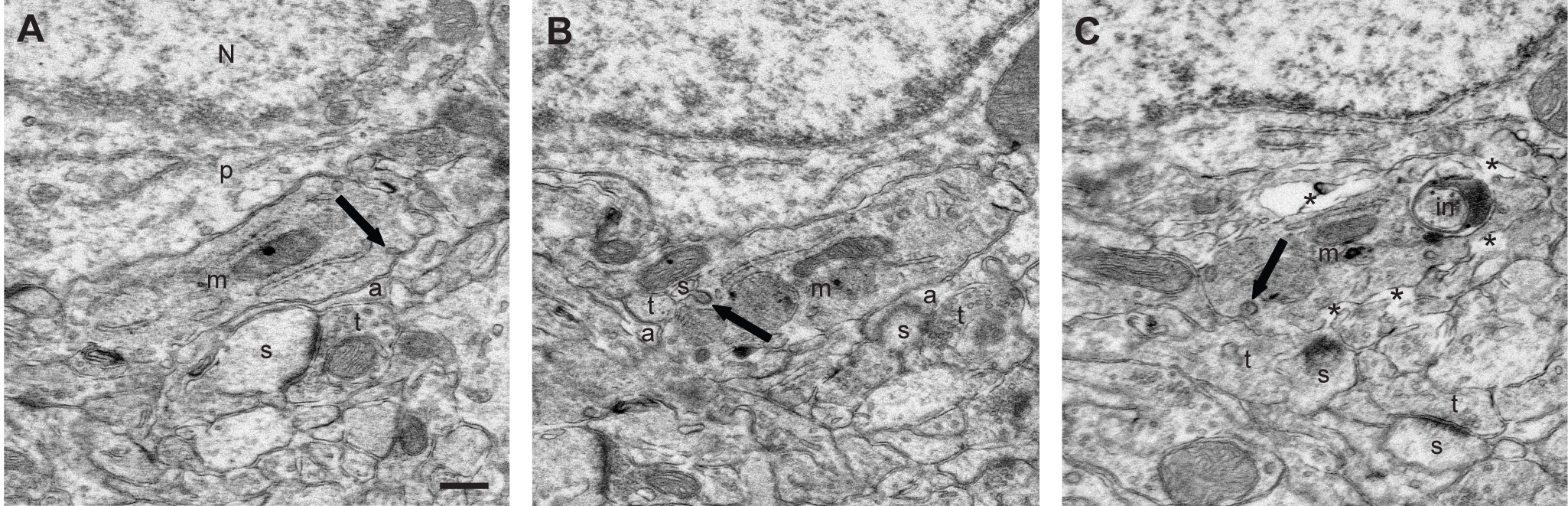

Supplement: Figure S3 — SSEM images showing additional examples of coated pits at the sites of cell–cell contact between microglia and synapse-associated elements. In these examples, the vesicles that appear coated (black arrows) are found inside a microglial process (m), at the sites of contact with an astrocytic process (a) (A), a dendritic spine (s) (B), and an axon terminal (t) (C). d, dendrite; N, nucleus; p, perikaryon. Scale bar = 250 nm. (2.05 MB TIF) [file pbio.1000527.s003.tif]

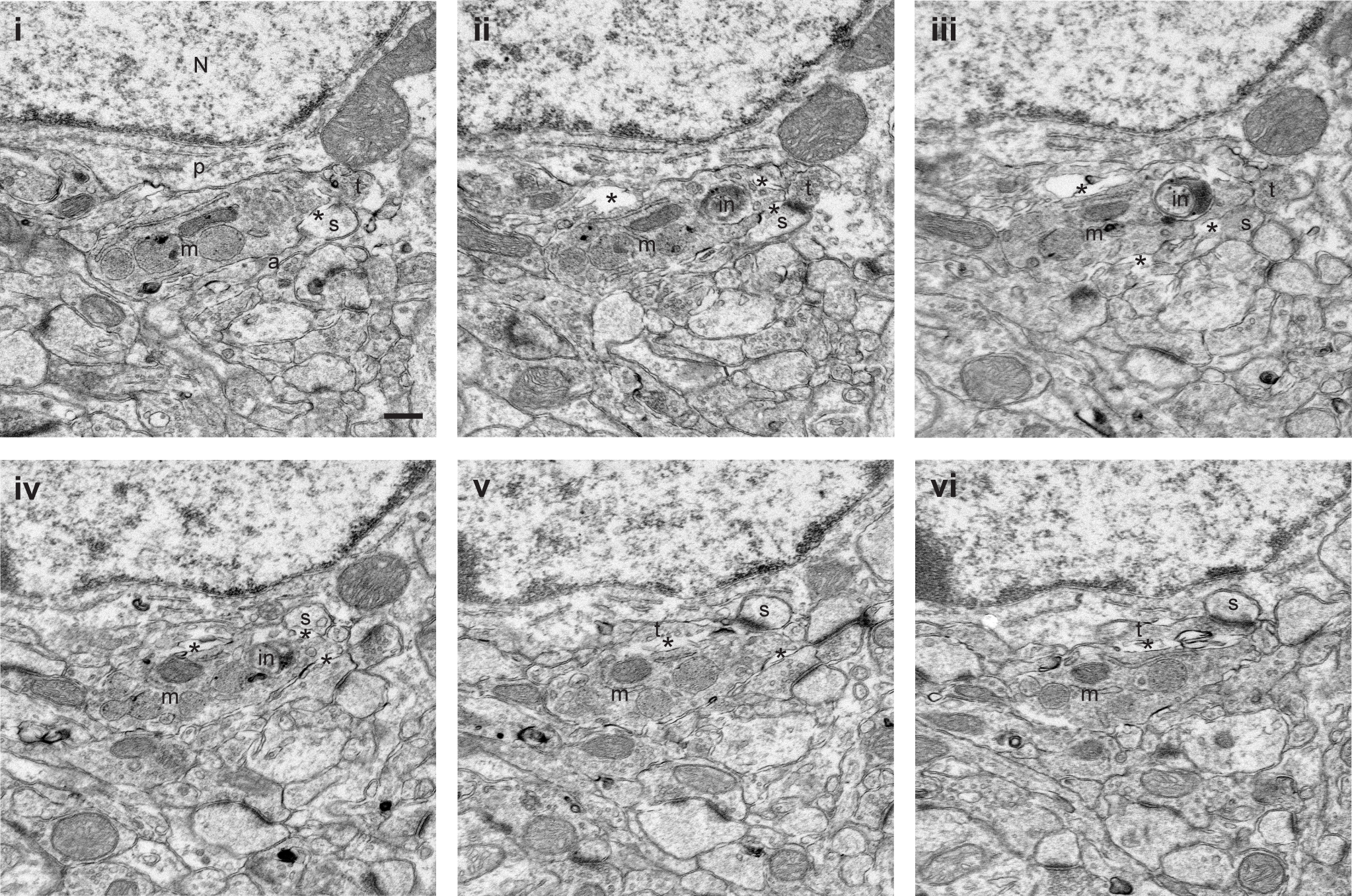

Supplement: Figure S4 — SSEM images from an animal undergoing normal visual experience, showing the phagocytic engulfment of cellular debris by a microglial process. Images are separated by 65 nm. *, extracellular space; a, astrocyte; d, dendrite; in, cellular inclusion; m, microglial process; N, nucleus; p, perikaryon; s, dendritic spine; t, axon terminal. Scale bars = 250 nm. (4.02 MB TIF) [file pbio.1000527.s004.tif]

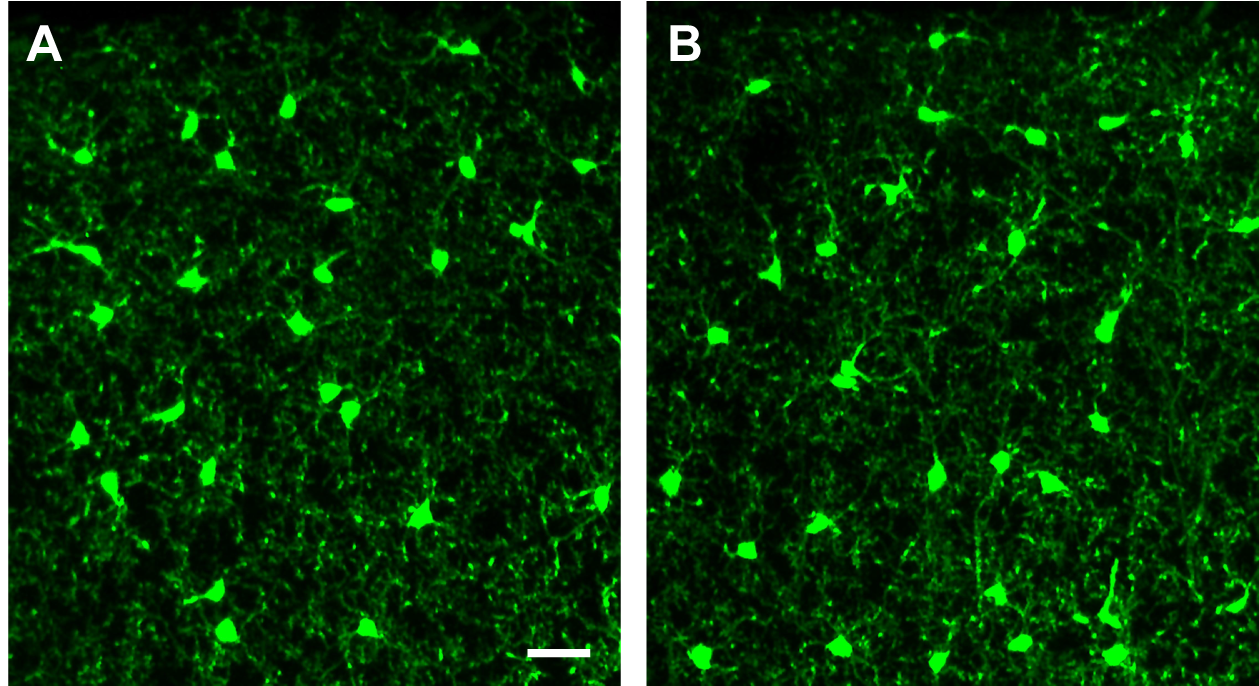

Supplement: Figure S5 — Z projections showing the morphology of microglia in brain sections of animals perfused after two-photon in vivo imaging. The imaged area is shown in (A), and the corresponding contralateral area is shown in (B). The pial surface is presented at the top of the image in both cases. The similar polarity, thickness, and density of microglial cell bodies and processes (green) confirm that microglia are not activated by transcranial imaging. Scale bar = 15 µm. (1.47 MB TIF) [file pbio.1000527.s005.tif]

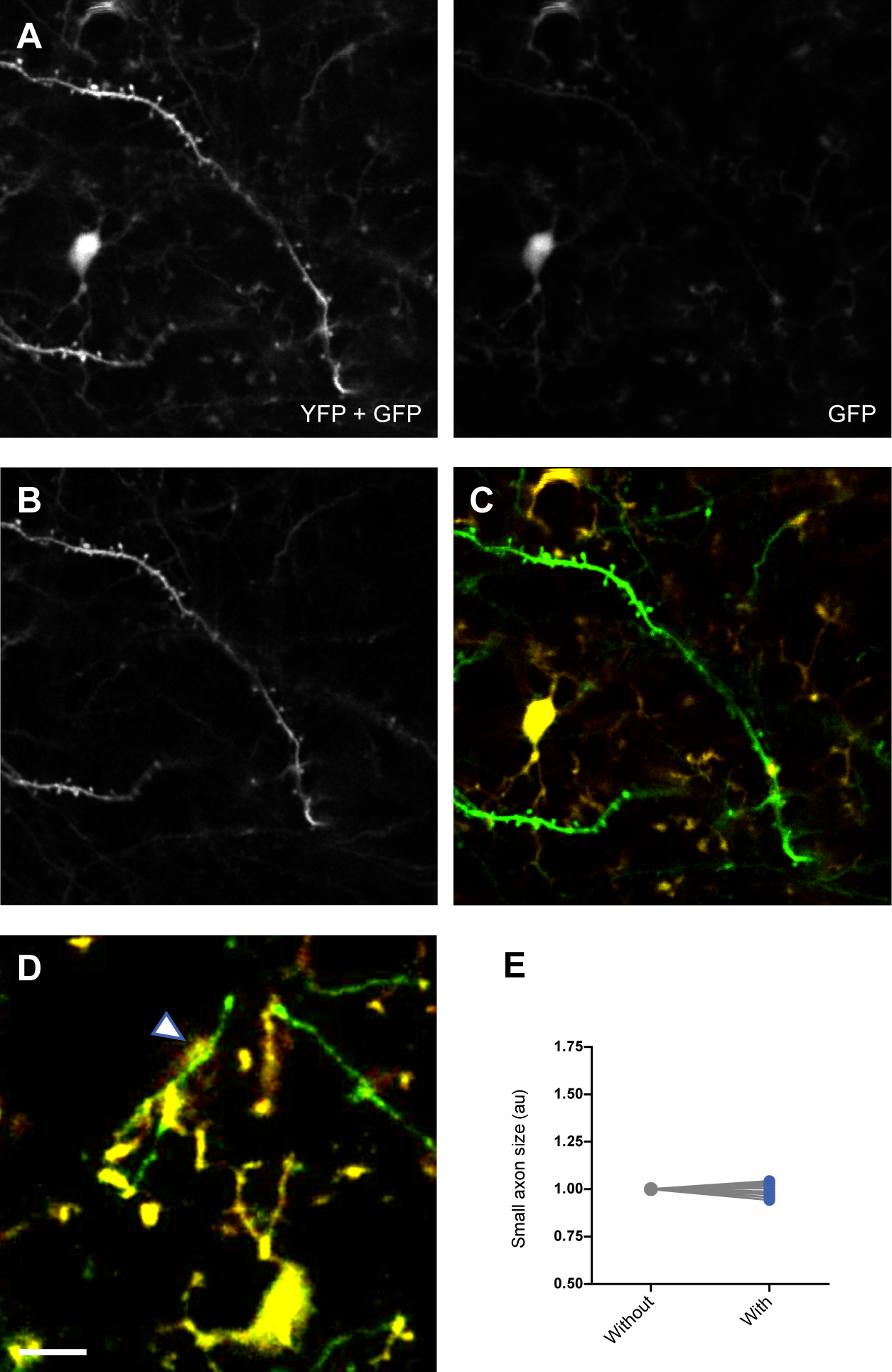

Supplement: Figure S6 — Separation of GFP and YFP fluorescence in CX3CR1-GFP/Thy1-YFP mice. (A) Two-photon images from the yellow channel (YFP+GFP; left) and green channel (GFP; right) in their uncorrected state. (B) Two-photon image from the yellow channel corrected with subtraction of background and GFP fluorescence for analysis of axon terminal and dendritic spine sizes. (C) Merge of the yellow and green channels (assigned the colors green and red, respectively) and adjustment of brightness and contrast for visualization of microglial contacts (yellow) with neuronal elements (green). (D) Two-photon image showing a contact between a microglial process (yellow) and a small axon (green; white arrowhead) during normal sensory experience. (E) Axon size without versus with microglia contact, normalized to the first condition for presentation purposes. au, arbitrary units. (1.42 MB TIF) [file pbio.1000527.s006.tif]

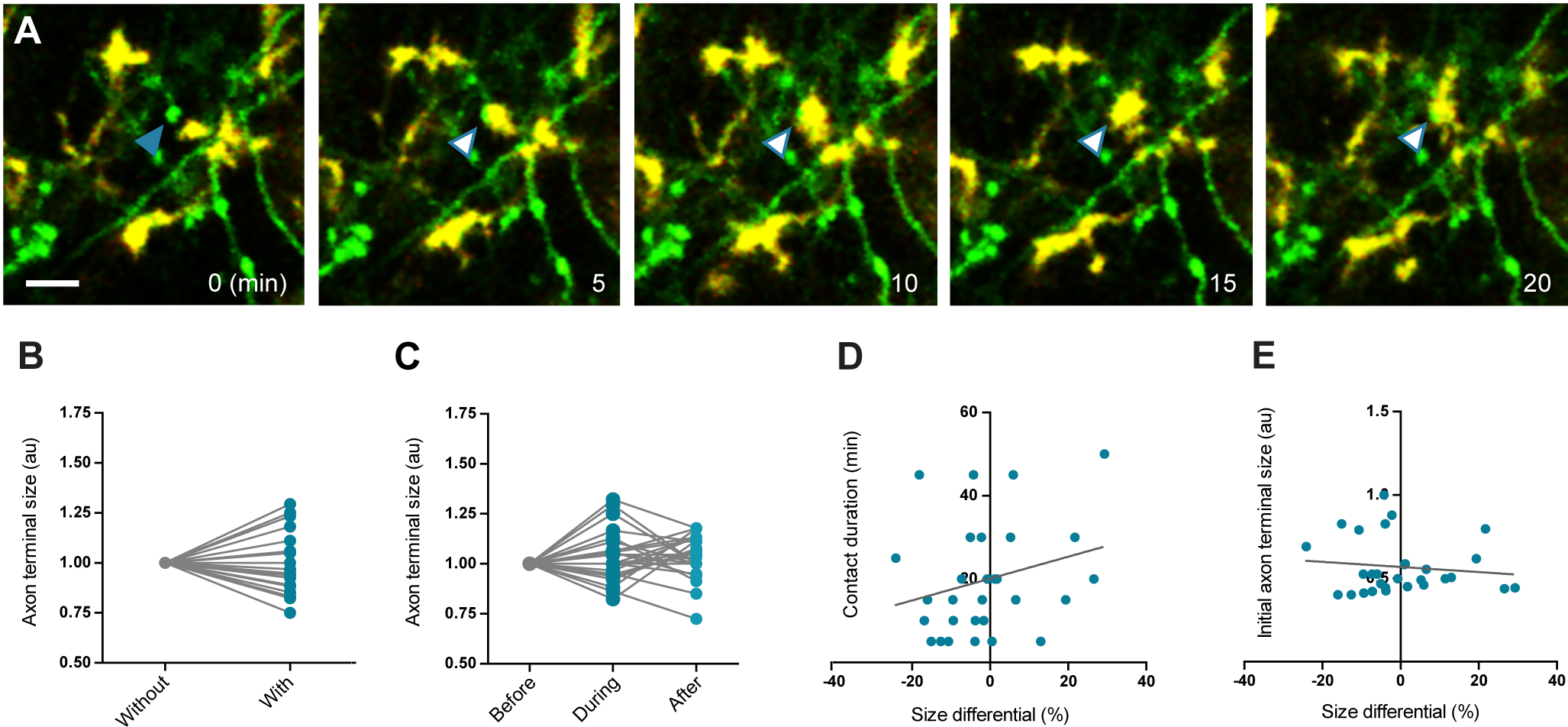

Supplement: Figure S7 — Structural/dynamic interactions between microglia and axon terminals during normal visual experience in vivo. (A) Time-lapse image showing an axon terminal (green; blue arrowhead) contacted by microglial processes with bulbous endings (yellow; white arrowhead) over 20 min. Scale bar = 5 µm. (B and C) Axon terminal size without versus with microglial contact (B) or before, during, and after contact (C), normalized to the first condition for presentation purposes. (D and E) Lack of correlation between microglial contact duration or initial terminal size (normalized to largest axon terminal) and the change in axon terminal size during microglial contact. au, arbitrary units. (1.17 MB TIF) [file pbio.1000527.s007.tif]

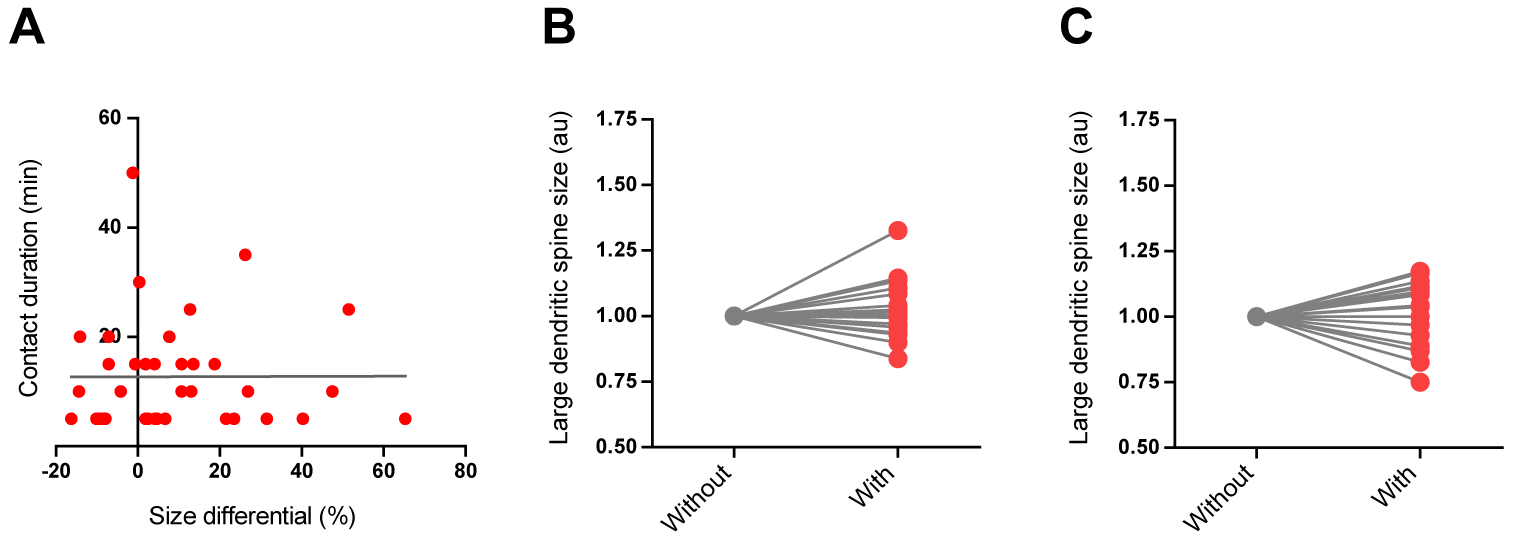

Supplement: Figure S8 — Additional analysis of structural/dynamic interactions between microglia and dendritic spines during normal visual experience in vivo. (A) Lack of correlation between microglial contact duration and the change in dendritic spine size during contact. (B and C) Change in the size of large dendritic spines assessed with the amplitude of the Gaussian fit to the fluorescent profile (as for other analyses of dendritic spine size presented in the Results section) or with the width of the fluorescent profile (1/e 1/2 radius of the Gaussian fit; see Materials and Methods section), confirming that the size changes of bigger spines were not underestimated with assessments of maximal fluorescence. (0.13 MB TIF) [file pbio.1000527.s008.tif]

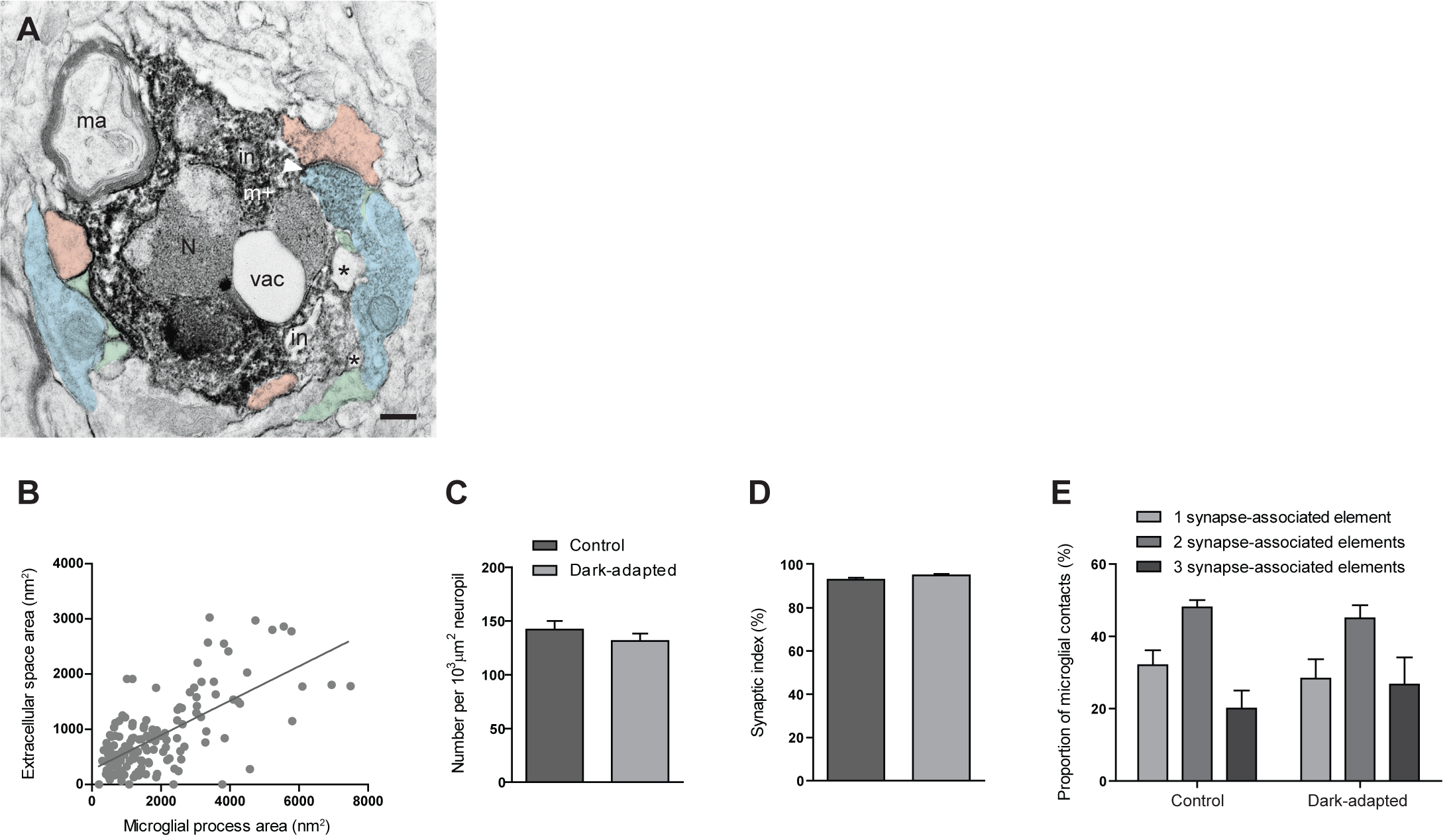

Supplement: Figure S9 — Additional analysis of ultrastructural interactions between microglia and synapse-associated elements during altered visual experience. (A) EM image taken in a DA animal showing a microglial (m+) perikarya that contains vacuole (vac) and cellular inclusions (in). *, extracellular space; ma, myelinated axon; N, nuleus. Scale bar = 250 nm. (B) Correlation between the areas of microglial processes and associated extracellular space in DA animals. (C) Total number of IBA1-immunopositive microglial processes in a surface of 1,000 µm2 of neuropil, in control versus DA animals (n = 3 animals per experimental condition; mean ± SEM). (D) Synaptic index in control versus DA animals (n = 3 animals per experimental condition; mean ± SEM). (E) Proportion of simultaneous microglial contacts with one, two, or three synapse-associated elements (n = 3 control and 3 DA animals; mean ± SEM). (1.17 MB TIF) [file pbio.1000527.s009.tif]

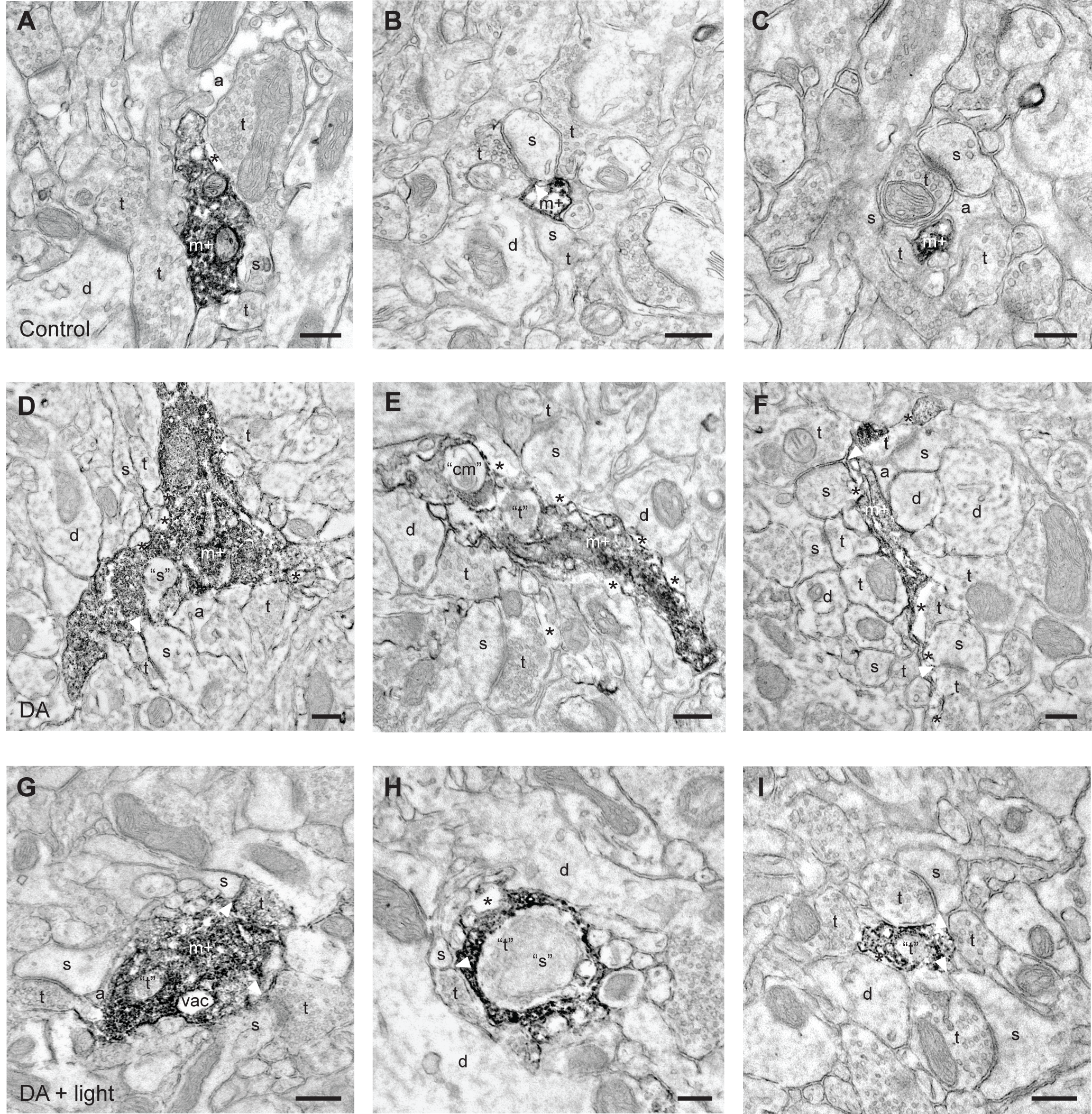

Supplement: Figure S10 — EM images showing additional examples of microglial processes contacting multiple synapse-associated elements, including synaptic clefts, in the different experimental conditions. a, perisynaptic astrocytic process; d, dendrite; m, microglial process, s, dendritic spine; t, axon terminal. White arrowheads indicate synaptic clefts. Scale bars = 250 nm. In (A–C; control animals), the small microglial processes (m+) are devoid of cellular inclusions and surrounded by narrow extracellular space. In (D–F; DA animals), larger microglial processes (m+) display bulky (D and E) or spindly (F) morphologies. While the bulky processes contain cellular inclusions resembling profiles of dendritic spine (“s”), axon terminal (“t”), or cellular membranes (“cm”), the spindly process is surrounded by extended extracellular space. In (G–I; DA+light animals), the large-to-small microglial processes display vacuole (vac) and cellular inclusions resembling terminals (“t”) as well as a synapse between a dendritic spine (“s”) and a terminal (“t”). Little extracellular space is observed. (7.32 MB TIF) [file pbio.1000527.s010.tif]

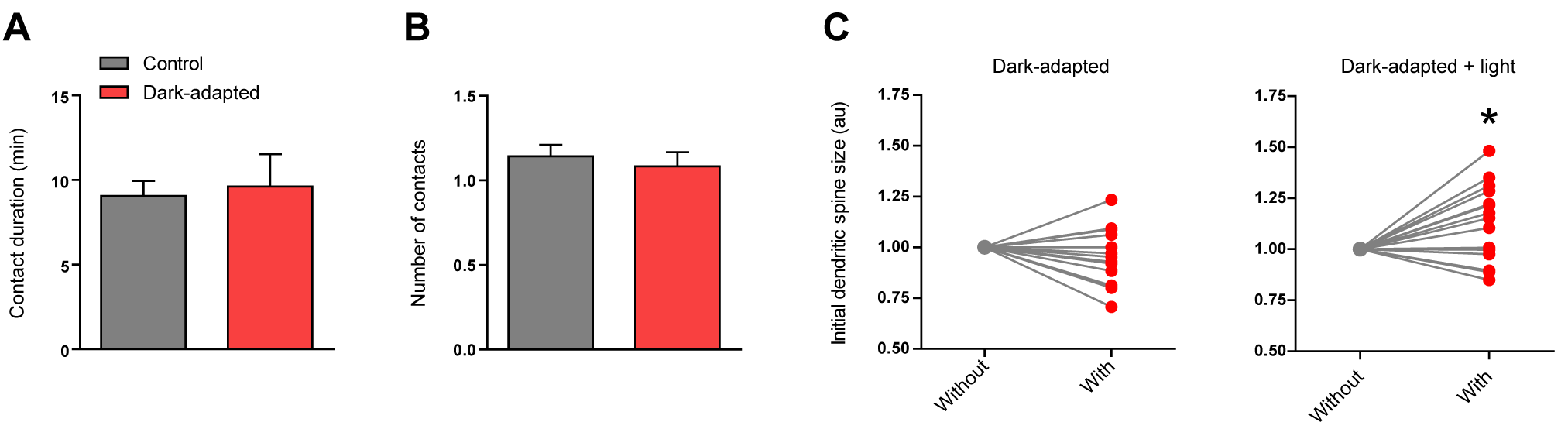

Supplement: Figure S11 — Additional analysis of structural/dynamic interactions between microglia and dendritic spines during altered visual experience in vivo. (A and B) Duration and frequency of microglial contacts with individual dendritic spines during 40-min imaging sessions, in DA and control animals (mean ± SEM). (C) Dendritic spine size without versus with microglial contact in DA animals (left) and DA+light animals (right), normalized to the first condition for presentation purposes. *, p<0.05. (0.14 MB TIF) [file pbio.1000527.s011.tif]

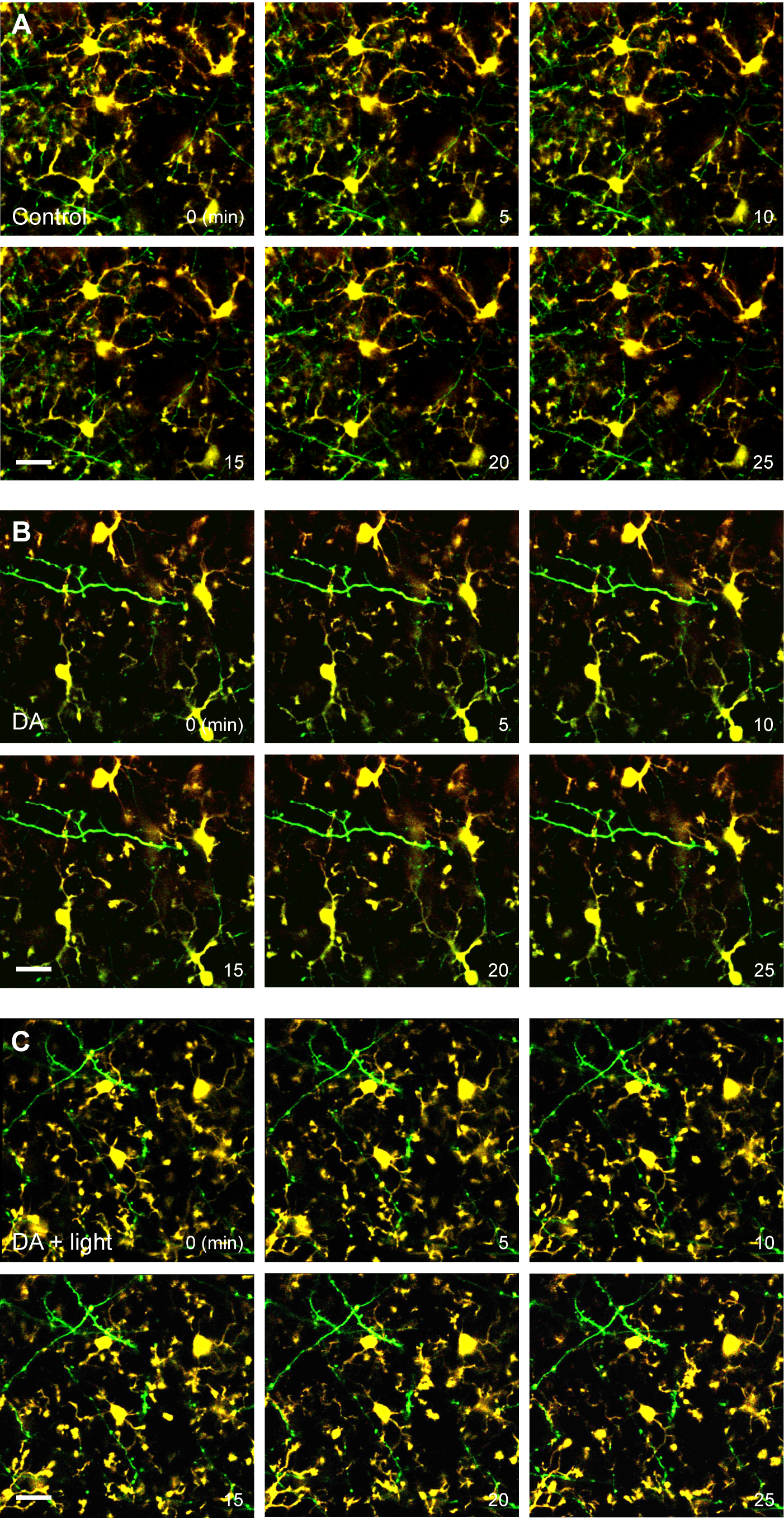

Supplement: Figure S12 — Time-lapse images showing additional examples of microglial morphology and motility in the different experimental conditions. Images from control (A), DA (B), and DA+light (C) animals are shown. In (B), note the thickening of microglial processes, which also are sparse. Scale bars = 10 µm. See also Videos S4–S6. (6.14 MB TIF) [file pbio.1000527.s012.tif]

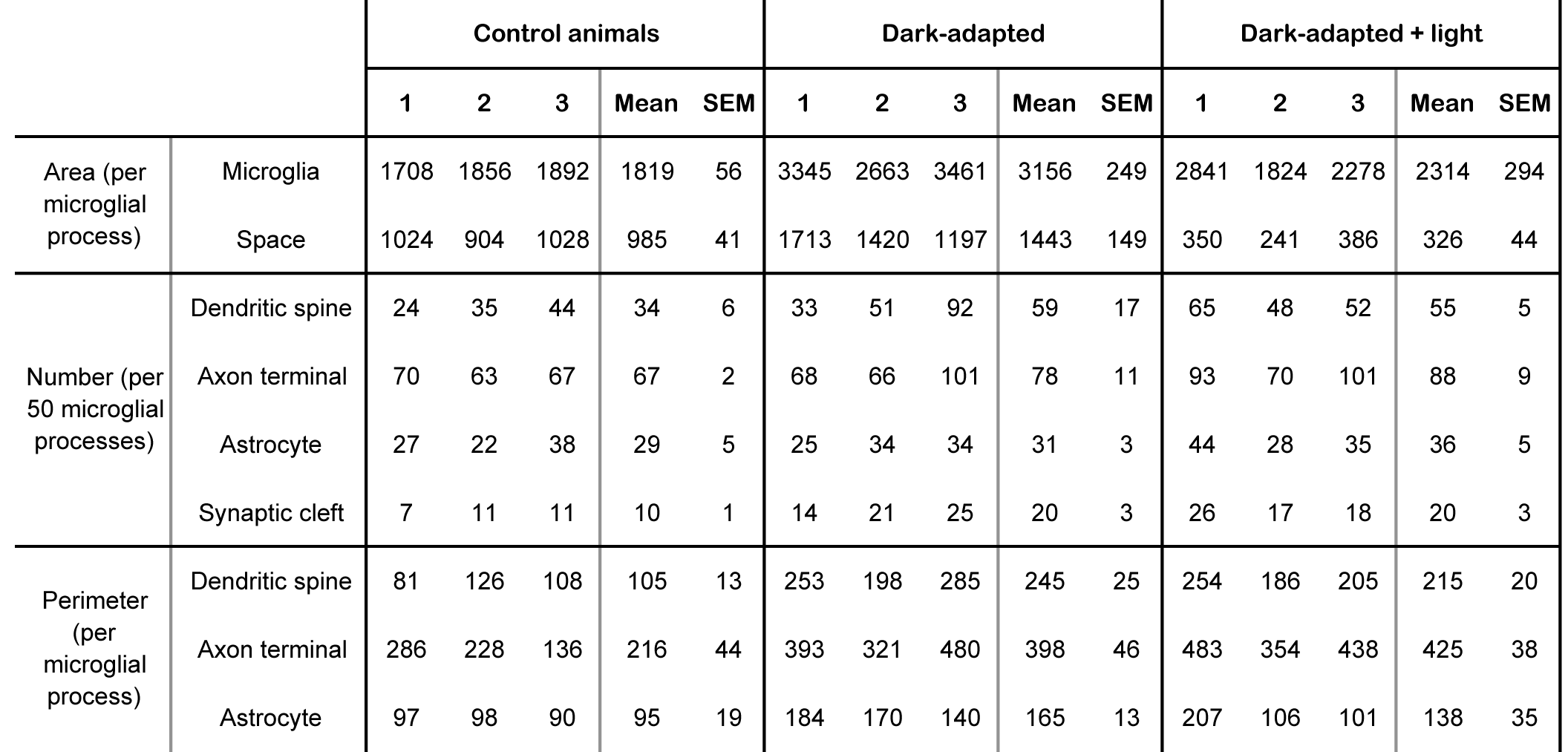

Supplement: Table S1 — Changes in the ultrastructural interactions between microglia and synapse-associated elements with visual experience. (0.21 MB TIF) [file pbio.1000527.s013.tif]

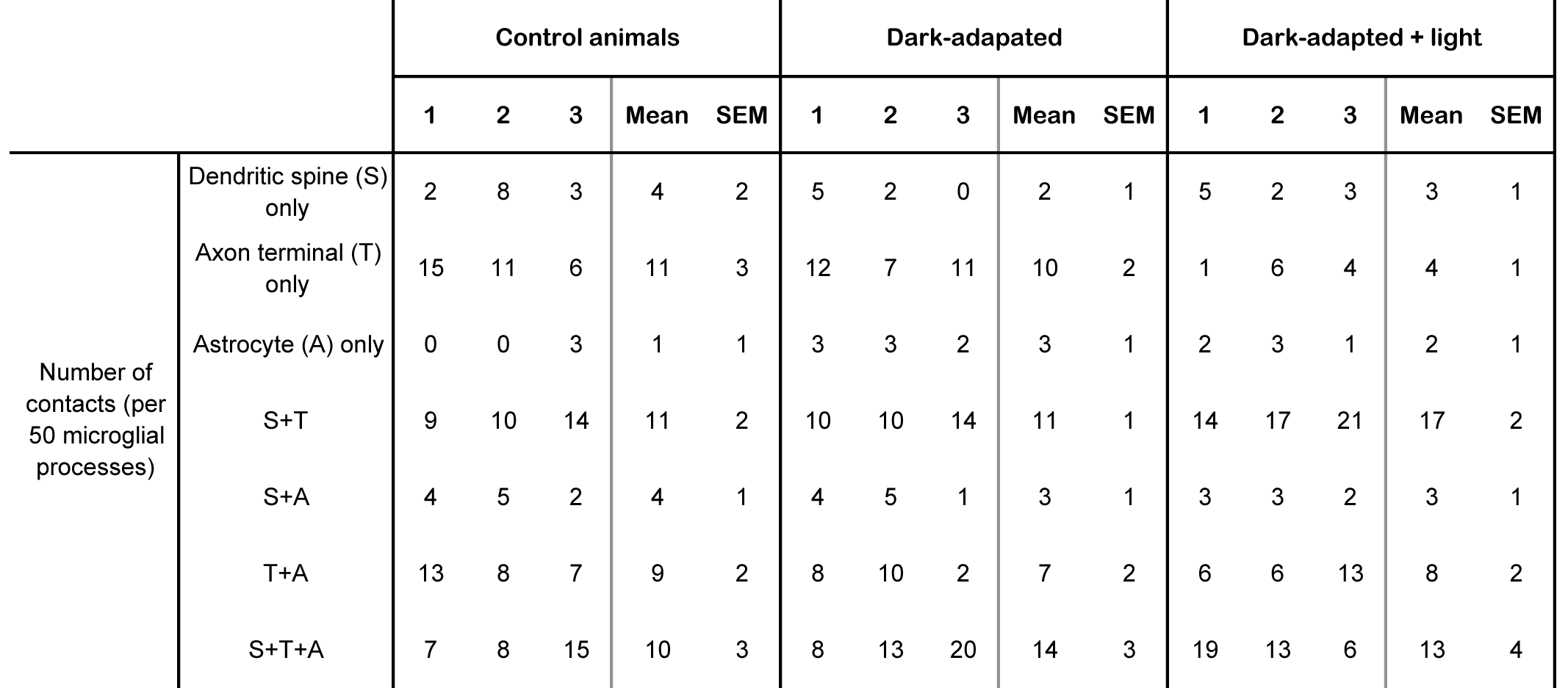

Supplement: Table S2 — Changes in the ultrastructural interactions between microglia and different combinations of synapse-associated elements with visual experience. (0.14 MB TIF) [file pbio.1000527.s014.tif]

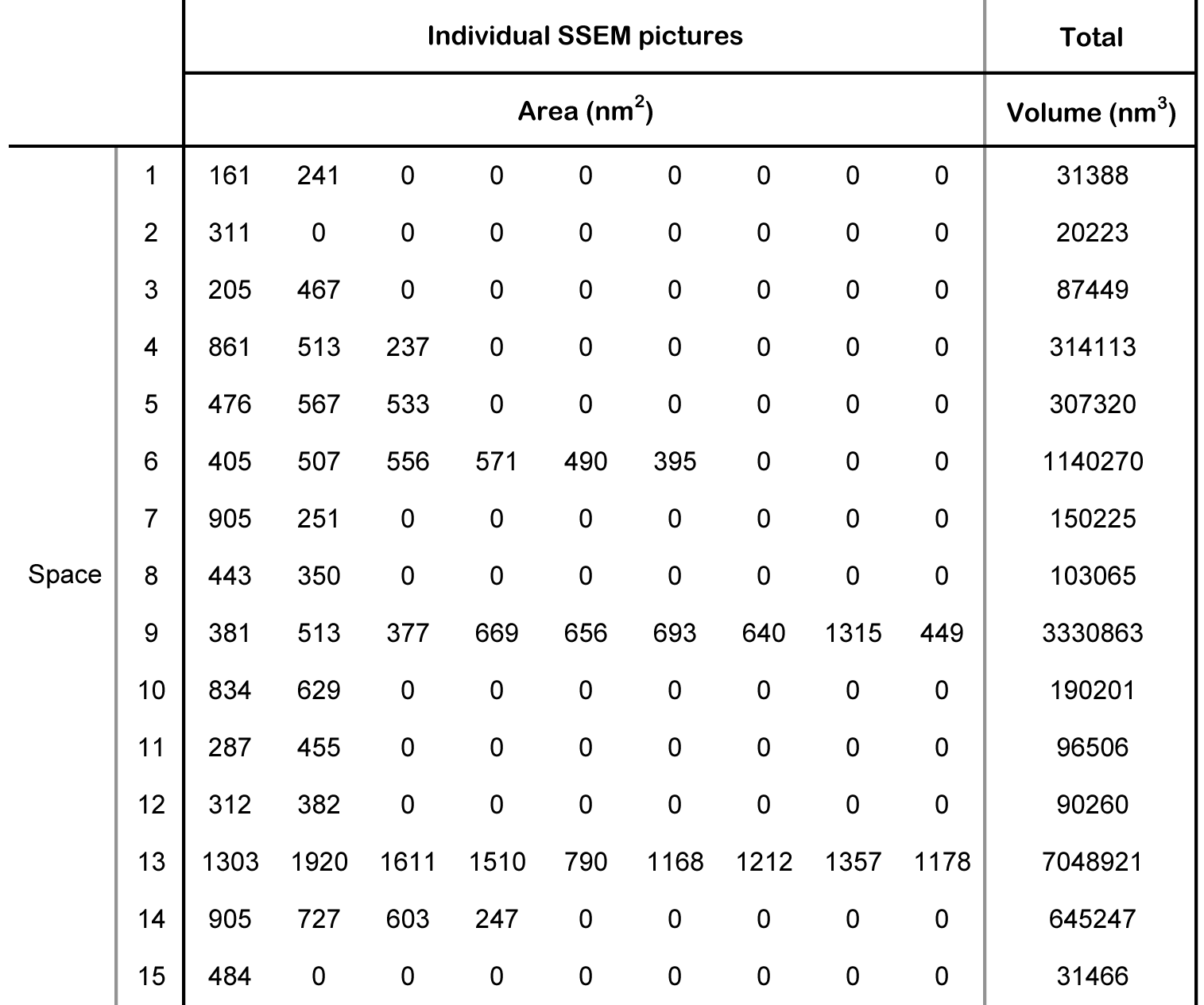

Supplement: Table S3 — Diversity in microglia-associated extracellular space volumes. (0.16 MB TIF) [file pbio.1000527.s015.tif]
